# Delhi urbanization footprint and its effect on the earth’s subsurface state-of-stress through decadal seismicity modulation

**DOI:** 10.1038/s41598-023-38348-7

**Published:** 2023-08-03

**Authors:** Deepak K. Tiwari, Manoj Hari, Bhaskar Kundu, Birendra Jha, Bhishma Tyagi, Kapil Malik

**Affiliations:** 1 Department of Earth and Atmospheric Sciences, NIT Rourkela, Rourkela, 769008 India; 2 Terrestrial Sciences Section, Climate and Global Dynamics, National Center for Atmospheric Research, Boulder, 80307 USA; 3 Department of Chemical Engineering and Materials Science, University of Southern California, Los Angeles, CA 90007-1211 USA; 4 Indian Institute of Technology (ISM), Dhanbad, Dhanbad, 826004 India

**Keywords:** Seismology, Geophysics

## Abstract

Urban land and its expansion have profoundly impacted the global environment, including the stress change in the earth’s subsurface, even though urban land is a small fraction of the global land surface. Divulging such effects has never been more important, given the role of stress in determining the safety of the urban population against earthquakes. However, knowledge of this time-dependent non-linear effect of urbanization on the subsurface remains in the gray area. This study focuses on the area surrounding Delhi, the capital city of India, to understand the relative contribution of the building load created by rapid urbanization in exacerbating the subsurface state-of-stress. The results highlight that, since 2010, the modulation in the seismicity rate and the stability of basement thrust faults is linked not only to urbanization but also to decadal groundwater storage. Mounting evidence suggests that the rapid urbanization, and the resulting non-tectonic horizontal compression, stabilize faults in the Aravalli Delhi belt, which are destabilized due to the extensive groundwater extraction. This affects the decadal seismicity trend around the Aravalli Delhi fold belt. Nonetheless, the magnitude of this time-dependent deformation influence on the seismicity modulation remains uncertain. The findings from this study quantify the geomechanical impacts of urbanization in the Delhi area for the first time.

## Introduction

### Urban land, population surge and subsurface state-of-stress

**Figure 1:**
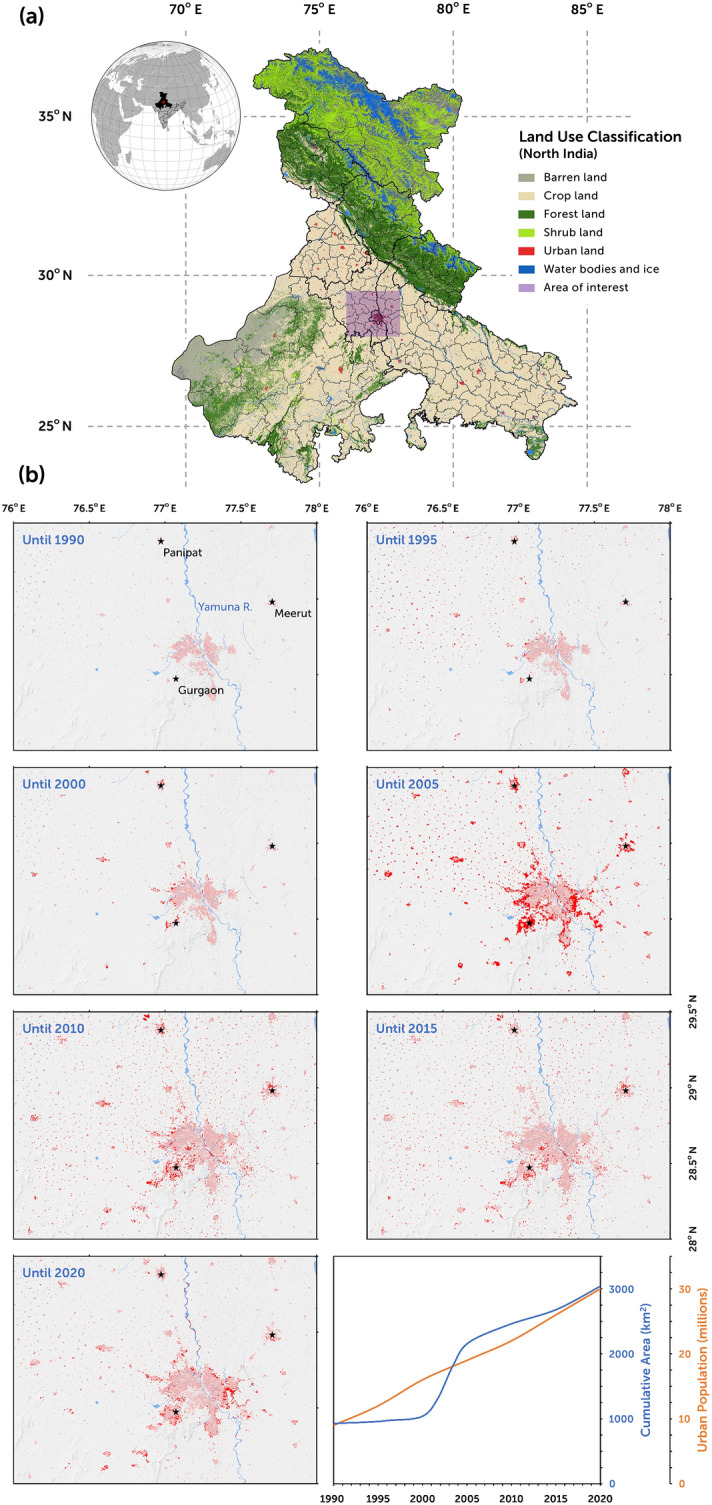
Land use map of the northern India region for the year 2020. (**a**) Land use classification (level I) of the northern India region for the year 2020, with the area of the study highlighted by a box. (**b**) Built-up land class transition during 1990–2020 with a time interval of 5 years. Pastel red color depicts the existing built-up area, and the solid red highlights the new land area included in the built-up class compared to the preceding time interval. The rightmost bottom panel shows the growth in the cumulative built-up land area and the urban population with time. This figure was generated using ArcMap (version 10.7.1; URL: https://support.esri.com.

Being a proximate nexus of environmental and anthropogenic interactions, urbanization reshapes various facets of the environment. As population growth guides rapid urbanization and aggressive metamorphosis of the earth’s surface, the atmosphere, hydrological cycle, ecosystem processes, climate system, and subsurface stability are projected to be disturbed dramatically at the local and global scales^1–4^. Urban lands occupy a small fraction of the global terrain and yet is home to 55% of the global population. Urban land area is accelerating faster than the urban population. Urbanization contributes to 70% of global warming and 80% of the loss of natural habitat. About 60% of the global croplands are in the proximity of urban areas, emphasizing the conflict between them^5^. Multiple studies^5–8^ highlight the proportionality between the rate of urban expansion and cropland diminution, with India, China, and Egypt being the focal examples (Fig. 1a, with Delhi and its surrounding regions in proximity to the cropland). In many developed countries, the population shift from rural to urban areas is projected to decrease, whereas the scenario is the opposite in the developing world, especially when the spotlight is over India. Angel et al.^9^ and Seto et al.^10^ projected that by 2030, the global urbanized land would be thrice that of 2000, in concert with a doubling of the urban population. In light of that, present-day urbanization is the genesis of mega-urbanization (coalesce of multiple urban fabrics forming a contiguous urban stretch^5^). Such mega-urbanization is forecast to occur in regions prone to high poverty rates, typically where agriculture rules the dominant sectors of the economy. Thus, an interdisciplinary understanding of how future urbanization and its population will alter the earth is paramount to alleviate the problem of global sustainability.

With the character of being an irrevocable land cover change (LCC), urban lands cast perpetual effects on their coupled environments. Holding strong altercations with the environment, urban expansion is the most significant anthropogenic factor responsible for altering the earth’s surface. The massive expansion of urban areas shifts the demographic trend, which not only alters the socio-economic pathways^2^ but also disturbs the biogeochemical cycles^11^, biodiversity functioning^12^, earth’s subsurface processes^4^, and hydrological systems^7^. Collectively, the literature points out that urbanization over time shapes the societal impacts of various climatic and environmental stresses. However, with the advancement of earth observation technologies via satellite remote sensing^11^, most of the urbanization-induced changes across various themes have been monitored. But, as most of the literature is confined to potential socio-environmental variables that are driven by urbanization, there is scarcely any understanding of how urbanization affects the earth’s subsurface stress regime and the potential for inducing (or triggering) human-induced earthquakes across different temporal and spatial contexts.

Human-induced earthquakes are increasingly becoming a matter of socio-political and scientific discussion in the past few decades, and more than 1200 cases have been reported in the HiQuake database, spanning the period from 1868 to 2022^13,14^. Hence, many researchers have explored the possibility of understanding and predicting human-induced earthquakes^15–19^. Dong et al.^20^ has proposed a tomography method for the identification of abnormal regions in complex rock-mass structures. From the travel time tomography and numerical and laboratory experiments, they were able to determine the size and strike of faults and also identify potential hazardous regions in underground geotechnical engineering sites^20^. Further, Dong and Luo^21^ argued that stress, velocity, material, fluid, and temperature have important ramifications for fault and earthquake mechanics. Barberio et al.^22^ investigated the groundwater responses to the worldwide seismicity and suggested that the Rayleigh waves from distant earthquakes cause variations in water table and as a result, modulated the nearby seismicity. The seismicity is also induced by hydrofracturing that has been used for extracting petroleum resources^23,24^. However, triggering or inducing earthquakes by changes in the surface load distribution due to anthropogenic and irrevocable LCC has been mostly overlooked. Such changes include manmade megastructures, skyscrapers, high-rise buildings, landfills, infrastructure constructions, and mining quarries. They are neglected due to their small dimensions relative to a typical geologic basin or a seismic study region. Relations between the rapid urbanization and the earth’s subsurface changes have long existed, albeit less well-quantified (except for a few reports, e.g., Lin.^25^; Klose^26^; Parsons^4^; Hu et al.^27^; Qian et al.^28^). Hence, little is known about the causative relationship between HiQuake and permanent LCC. Unraveling this link is critical for better seismic hazard assessment, mitigation of induced earthquakes, and the characterization of the state-of-stress of the seismogenic faults, specifically in response to building infrastructure construction. This motivated us to quantify the changes in the subsurface state-of-stress due to the dynamics of urban land expansion so we can better understand the consequences of these changes.

Our analysis is grounded in an internally consistent land use classification (LUC) analysis framework that utilizes 30 years of satellite data by creating LUC maps at a 5-year interval from 1990 using the Landsat data and partitioning urban land area around the Delhi region of Northern India which includes India’s heavily populated capital city, Delhi. The LUC dynamics across Delhi and its surroundings (boxed area in Fig. 1a) is derived from the reference data. We quantify LUC dynamics and its associated uncertainty at the local scale to maintain the accuracy and resolution of our analysis. We quantify the spatial weight distribution in the Delhi surrounding region to reveal the cumulative impact of stress change on the stability of the subsurface faults. We also argue that the weight distribution has links to the decadal seismicity modulation around the Aravalli Delhi fold belt due to increasing and irreversible mass redistribution from the ground settlement, subsidence, groundwater extraction, and seasonally controlled hydrological loading cycle.

## Results and discussion

### Urban land cover change and accuracy assessment

It has been observed that the LCC around the Delhi and surrounding region evolved with time at different rates in different epochs (Fig. 1b). The maximum LCC was observed during 2000–2005 when the ‘crop land’ class had a significant reduction in area, and the ‘built-up’ class had a relentless surge (described in Methods). Similarly, the ‘forest land’ class experienced a large reduction in the area during the epochs of 1990–1995 and 2000–2005. Overall, urbanization continued to increase, and the crop land and forest land areas continued to decrease. Since our study is about urbanization effects, we chose to focus our interpretation on the ‘built-up’ class. A consistent LUC map for the urban surge at a 5-year epoch from 1990 to 2020 was developed for Delhi and its surrounding region and is presented in Fig. 1b. The intensity of ‘built-up area’ LCC was comparatively stable from 1990 to 2000 with an intensity of 2.67% (± 0.95%). Later, the LCC area had a relentless growth during 2000–2005 (Fig. 1b, rightmost top panel) with an intensity of 69.13% (± 23.72%) in contrast to its value in 1990, indicating that the urbanization had an unmatched shift (Fig. 1b, rightmost bottom panel). But the change in intensity was restrained later to stay within the range of 6–9% from 2005 to 2020, as depicted by a slower rise in the LCC cumulative area curve during these epochs. From the temporal perspective, the decadal transition of the urbanized area per grid cell (per 100 km^2^) in decade 1 (1990–2000) showed the lowest slope of 12.34 (± 7.63) km^2^ per 5-year epoch, whereas the maximum slope is observed for decade 2 (2000–2010) with the highest slope value of 87.75 (± 31.43) km^2^ per 5-year epoch. For decade 3 (2010–2020), the slope is 24.49 (± 12.55) km^2^ per 5-year epoch. From the spatial front, urbanization had a leapfrog sprawl during 2000–2005, mostly over the Delhi buffer. Prior to this, the region had outlying sprawl (new regions were not in direct spatial connection with the Delhi region) from 1990–2000. Post-2005, the study region mostly experienced the edge expansion (new regions in direct contact with the edges of the Delhi region), which was observed with periodical shifts (i.e., the prominent 2005 urban sprawl continues to expand south-west in terms of edge sprawl prior to 2015, which shifted to south-east in the 2020 epoch).

**Figure 2:**
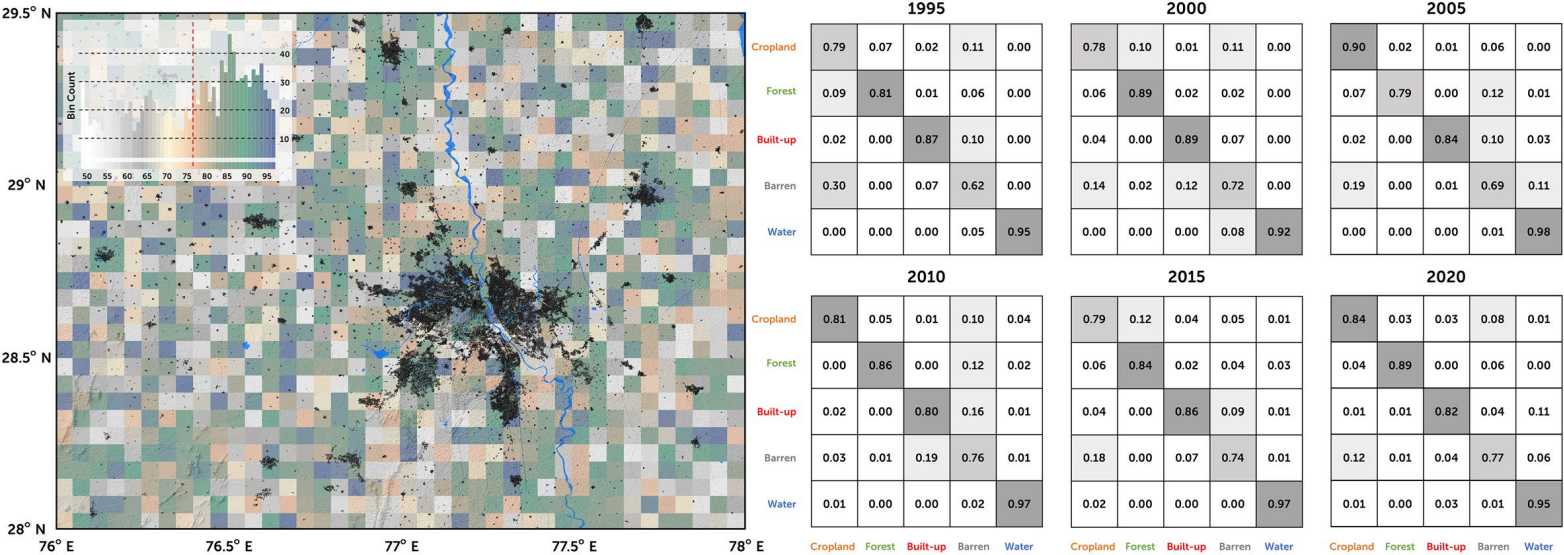
Land use classification accuracy plot for the period of 1995–2020. The left panel highlights the classification accuracy for a 5 × 5 km grid, aggregated since 1990. The inset map plots the accuracy scores of the grid cells. The normalized confusion matrices in the right panel highlight the classification accuracy and the classifier bias for the corresponding years (the confusion matrix for the year 1990 is similar and not shown to maintain the figure shape). This figure was generated using ArcMap (version 10.7.1; URL: https://support.esri.com).

Computing the percentage of the overall agreement, or the percentage of effective agreement, is an implicit method to determine the agreement among the classified variables in the LUC science. While the accuracy assessment can show how much agreement exists among the variable classes, they do not factor in the possibility of the agreement by chance alone. Valid agreement among the variable classes exists only when the level of the agreement exceeds what would be expected by chance alone. In the context of improving the confidence in our classification system, one such true agreement method—Cohen's kappa coefficient (κ)—was used to assess the statistical stability and the interrater reliability among the classes. With that, the overall accuracy of the LUC was mapped to the range of 79.81–91.76%, with the maximum accuracy observed for 2010 (with κ of 0.93) and the minimum accuracy observed for 1995 (with κ of 0.81) (described in Methods). Accuracies across the LUC classes were heterogeneous (e.g., cropland had a lower mean accuracy of κ = 0.79, and the built-up class has a higher accuracy of κ = 0.96). Though the pattern fluctuated across the classes, the accuracy of the built-up class was observed to have a consistently higher degree of κ. This high degree of accuracy across the study region can be inferred from Fig. 2. Despite higher levels of agreement between the classes, misclassification between the barren land and built-up class existed due to the confusion in spectral allocation. However, this does not affect the overall nature of the results because the temporal coverage of the built-up class tends to have the maximum accuracy with less confusion (Fig. 2, confusion matrix panels) in reference to the spatially gridded area (Fig. 2, gridded panel). Based on the temporally stratified matrix, the year 2000 had the maximum accuracy for the built-up class by sharing confusion with the barren land class. The minimum accuracy was in the year 2010 when the built-up class shared its confusion with the barren land and cropland classes. Thus, the higher overall accuracy and explicit class-wise accuracy (especially for the built-up class), with negligible uncertainty across spatial and temporal scales, indicate the validity of the urbanization trend over Delhi and its surrounding region. This provides the necessary confidence in using the urbanization map to calculate the effect of urbanization on the subsurface state-of-stress.

### Weight of the urbanization, groundwater storage change, and ground subsidence

Quantification of the weight of the urban areas is virtually impossible due to a large number of individual structures of variable size and shape in urban lands. Import and export of goods also lead to mass flux in urban areas. We approximate the weight of the urbanization under the assumption that buildings and their content make up the dominant part of the weight, i.e., weight contribution from residential/commercial buildings, public buildings, light and heavy industrial buildings, warehouses, transportation centers, etc. In the absence of an open archive (or public database) of building outlines and specific heights of individual buildings, we considered our urbanization footprint map as a direct proxy for building outlines for computing the effects of urbanizations on the subsurface state-of-stress over the Delhi surrounding region. According to the Indian Standard Code of Practice for Design Loads (other than earthquakes) for Buildings and Structures^29^, the building load is categorized into dead load and live load. The dead load consists of the structural load of the building (ceiling, flooring, and walls). The live load includes all building contents such as people, food, water, furniture, interior utilities, parked vehicles, etc., as well as the load due to impact, vibration, and dust/debris, but it excludes load due to wind, seismic activity, and snow. The dead load value of building components ranges within 0.25–0.74 kN/m^2^ for a ceiling thickness range of 13 to 25 mm and 1.18–1.96 kN/m^2^ for a flooring thickness range of 100 to 125 mm, assuming concrete as the primary building material used for construction^29^. According to the Indian Standard of building code, the dead load of a single-floor building does not exceed 5.0 kN/m^2^, and this value increases with the increasing number of floors in a building^29^. Further, the live load value of the building ranges from 5.0 kN/m^2^ for a light industrial building to 10.0 kN/m^2^ for heavy-duty, while the other buildings, such as residential houses, schools, shops, and malls, have ranged within 3.0–5.0 kN/m^2^ as per the Indian Standard^29^.

**Figure 3:**
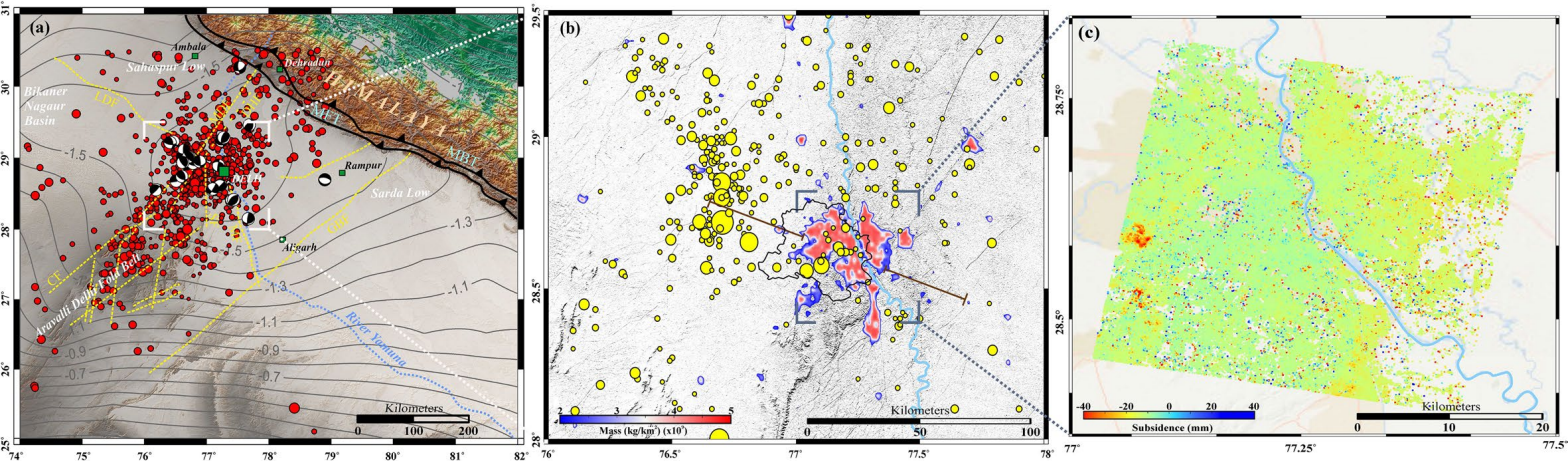
Groundwater extraction, cumulative area changes and ground subsidence from the Delhi surrounding region (**a**) Rate of groundwater depletion in the region derived from GRACE and GLDAS (contours in cm/year). Note the Aravalli Delhi fold belt associated seismicity (marked by the red circles in (**a**)) lies in the region of maximum groundwater depletion (~ 1.6 cm/year). Beach balls represent 15 significant earthquakes’ focal mechanisms. Different basement faults are marked by yellow dashed lines, MDF Mahendragarh Dehradun Fault, CF Chahapoli Fault, SF Sohna Fault, MF Mathura Fault, GBF Great Boundary Fault, DHF Delhi Hardwar Fault, MBT Main Boundary Thrust, MFT Main Frontal Thrust. (**b**) The cumulative area changes from 1990 to 2020 around Delhi is summed over 1 km^2^ grid, and the estimated mass of buildings is distributed over the surface. The brick red line represents the cross-section across the Yamuna river used to estimate line load for Coulomb failure stress change ($\Delta CFS$) calculation. The black line represents the state boundary of Delhi. (**c**) The ground subsidence in New Delhi, National Capital Region (NCR) is analyzed using multiple SAR sensor data and the PS-InSAR technique^30^. The plot represents the ascending Cosmo-Skymed vertical cumulative displacement acquired from June 8, 2011 to November 15, 2017 over Delhi. The (**a**, **b**) was generated using Generic Mapping Tools (version 6.3.0; URL: https://www.generic-mapping-tools.org/download/) and (**c**) was generated using ArcMap (version 10.7.1; URL: https://support.esri.com).

Since it is difficult to count all individual loads and their type (dead or live), we assumed 5.0 kN/m^2^ load as the standard value combining dead and live contributions for calculating the weight of a city. Hence, our results should be considered minimum estimates. We computed the cumulative mass (in kg) of a building footprint over the Delhi surrounding region by combining the live and dead load contributions (i.e., 5.0 kN/m^2^), dividing by the gravitational constant (g = 9.81 m/s^2^) and finally multiplying by the cumulative built-up area (Fig. 3b). The urban load distribution has been computed by considering building weight at their centroid points, dividing the study domain into 1 × 1 km^2^ cells, and subsequently summing the weight from all centroids that occur within a cell. As expected, the maximum weight concentration occurs in and around the urban built-up areas of the Delhi surrounding region along with specific hotspots locations, e.g., Gurgaon, Meerut, Yamuna riverbank areas, New Delhi International Airport and adjacent region, where there are multiple skyscrapers and tall/heavy buildings occupying relatively small areas.

Being the capital city of India, Delhi is heavily populated and, hence, has experienced overexploitation of groundwater aquifers due to extensive irrigation, urban development and expansion, and low/fluctuating rainfall due to the global climate change scenario. As a result, the region has suffered significant ground subsidence in the past few decades. In order to quantify the groundwater extraction rate in north-western India and the Delhi surrounding region, we have explored Gravity Recovery and Earth Climate Experiment (GRACE) data and Global Land Data Assimilation System (GLDAS) (described in Methods). From this, we estimate the rate of groundwater storage change as ~ 1.6 ± 0.6 cm/year during the 2002–2015 period, which is alarming in the Delhi surrounding region (Fig. 3a, contours in cm/year). Another interesting point to note is that seismicity in the Aravalli Delhi fold belt region is possibly linked with the paleo-structure^31^ and lies in the region of maximum groundwater depletion (~ 1.6 cm/year). Although the entire Aravalli Delhi fold belt has witnessed several moderate-to-strong historical earthquakes^32,33^, the Delhi region appears to be more active seismically, and several small magnitude earthquakes have been recorded by a dense seismic network operating in the region (Fig. 3a). The majority of these earthquakes occur in the upper crust, within ~ 25–30 km depth, and a majority of them involve reverse fault motion along with some strike-slip component on steep fault planes dipping ~ 50–65°^33^ (Fig. 3a). The Delhi surrounding region is covered by alluvium, which is occasionally traversed by the linearly elongated quartzite ridges of Proterozoic age, which act as a basement rock and also host several basement faults^34,35^.

An important observation is that the estimated ground subsidence from multi-sensor radar data around the Delhi surrounding region appears to follow the urbanization footprints (Fig. 3c. described in Methods). Ground displacement observations from the Cosmo-Skymed data have captured the subsidence rate at ~ 2–18 mm/year. We noted a higher rate of ground subsidence at ~ 15–18 mm/year in and around several locations, e.g., Gurgaon, Saraswati-Vihar, Surya-Vihar, Dundahera, Ashok-Vihar, Faridabad, and the eastern side of Yamuna river that includes Noida, Ghaziabad, and Shahdara. Further, the area closest to the New Delhi International Airport and adjacent regions of Prahladpur-Dwarka (covering ~ 15 km^2^ area) appears to be subsiding at a substantially higher rate of ~ 15–35 mm/year, possibly due to dense and heaviest building construction along with the extensive withdrawal of groundwater. The observed ground subsidence rate around the Delhi surrounding region appears to be much higher than the predicted ground subsidence rate (~ 2 mm/year) from a coupled hydro-mechanical model that simulated decadal groundwater extraction^36^. Also, the observed ground subsidence rate appears to be much higher than the subsidence rate estimated from the sparsely located geodetic (cGPS) data and the subsidence rate predicted by the LSDM hydrological model of the Delhi surrounding region (Figure S1) (~ 1–3 mm/year).

### Effects of urbanization on the subsurface state-of-stress

Besides these indirect effects, urbanization in the Delhi surrounding region (or the weight of the city) can be directly linked with the subsurface state-of-stress and basement fault stability in the Aravalli Delhi fold belt, which eventually modulates moderate and strong magnitude earthquakes during the seismic cycle. The anthropogenic mass accumulation in response to rapid urbanization in the Delhi surrounding region did not happen instantaneously but rather grew as the urban population did (Fig. 1b). Hence, the loading response of the crust is not instantaneous and ramps up with time. The elastic parts of the crust respond instantaneously to the urbanization load, and the time history of loading is not particularly significant for the elastic deformation response. However, near-surface geologic layers are more ductile, and rocks beneath the elastic upper crust deform nonlinearly, which makes their response time-dependent even if the loading is independent of time. In the following section, we quantify the effect of urbanization on the subsurface state-of-stress considering the above two aspects and excluding the aspect due to the viscosity structure of the subsurface, which is likely much more complicated.

### Impact of the urbanization weight on the elastic crust stability

An increase in the vertical stress due to urbanization weight on the ground, brittle failure under the vertical loading, and changes in the basement fault stability are highly sensitive to the nature of fault planes and faulting mechanisms. Brittle failure under vertical loading conditions would be favored in a normal faulting mechanism with steeply dipping faults. Unloading due to natural erosion, extraction of fluids from the basement^37^, or anthropogenic mass removal promotes a thrust faulting mechanism. Stress inversion of the available focal mechanism solutions from the Aravalli Delhi fold belt region suggests a stress orientation state in which the maximum principal compressive stress is in the NNE-SSW direction with a moderate plunge, i.e., identical to the stress orientation in the adjacent Garhwal-Kumaun Himalaya. Hence, the rate of seismicity production is expected to decrease under anthropogenic loading conditions.

**Figure 4:**
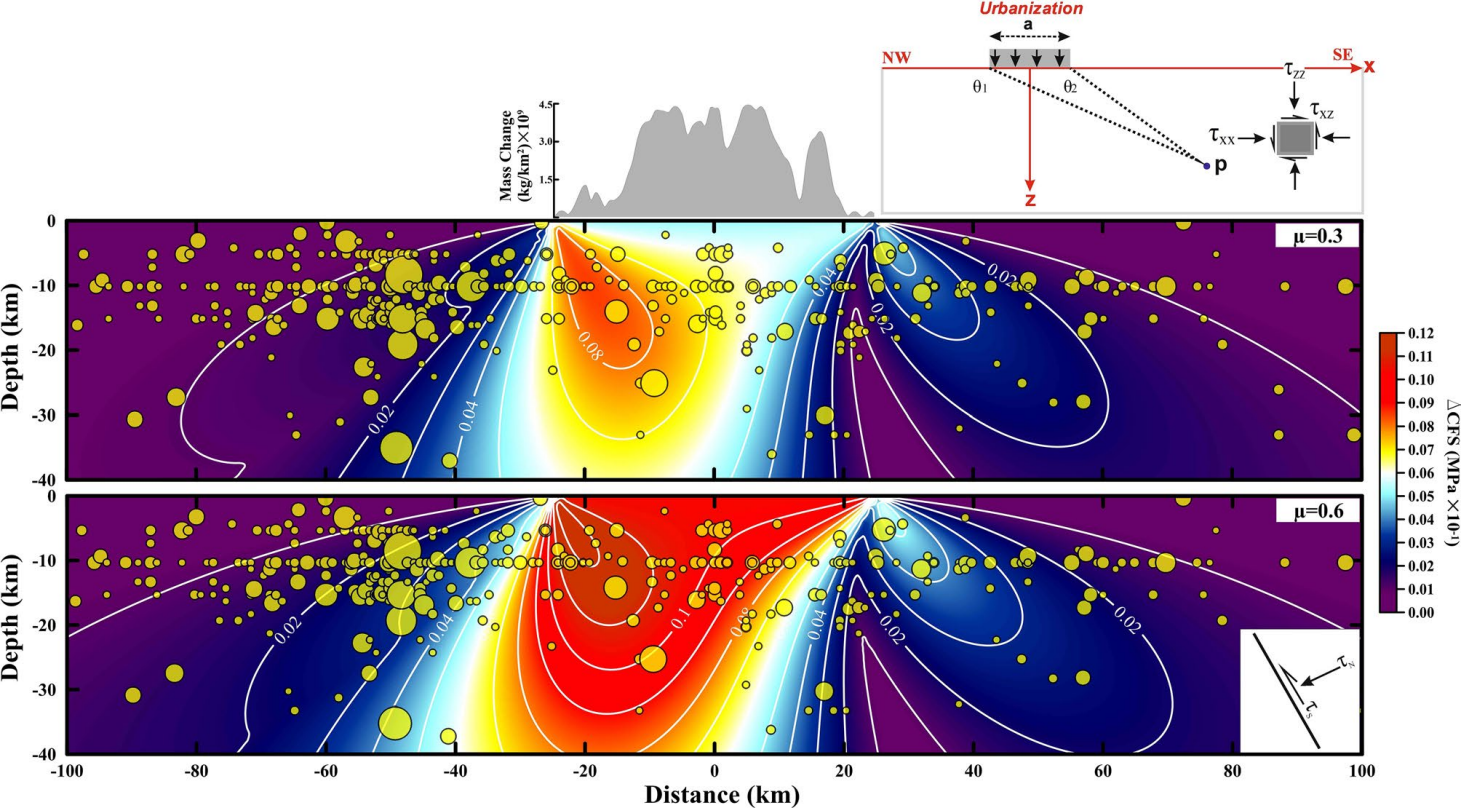
Coulomb failure stress change due to urbanization load. The change in Coulomb failure stress ($\Delta$CFS) due to urbanization load (building load over an area) plotted on a vertical section along a horizontal profile (marked in Fig. 3) across Delhi. Upper and lower plots are for friction coefficient values of 0.3 and 0.6, respectively. $\Delta$CFS (MPa) is resolved on the subsurface fault dipping at 60°. The model setup is represented in the top right corner. The line load acting on the elastic half-space is distributed over width *a.* θ_1_ and θ_2_ measure the angle downward from the positive x-axis to any point *P* at depth. The depth-wise distribution of earthquakes with magnitude (Mc > 2.5) observed from 2000 to 2020 in the study area. This figure was generated using Surfer graphical application (version 13.6.618 URL: https://www.goldensoftware.com/products/surfer) and Grapher graphical application (version 16.6.478 URL: https://www.goldensoftware.com/products/grapher).

To assess this while including urbanization-induced loading in the Delhi surrounding region, we computed the rate of Coulomb failure stress change (ΔCFS) on a basement fault due to the urbanization weight estimated earlier. Stress components (${\tau }_{xx}$, ${\tau }_{zz}$, ${\tau }_{xz}$) at any point (*P*) at a specific depth, resulting from a vertical load (${N}_{0}$ in N/m) within a homogenous elastic half-space, are represented as functions of the geometrical position of the load expressed in terms of angles θ_1_ and θ_2_ from both edges of the load (angles measured clockwise from the positive *x*-direction), the width of the load ($a$), and the load value ${N}_{0}$ (Fig. 4, top inset panel) (described in Methods^38^). ${N}_{0}$ is negative for unloading and positive for loading, and the *z*-axis is positive downward. Shear traction (${\tau }_{s}$) and normal traction (${\tau }_{n}$) components can be resolved on a basement fault plane dipping at an angle $\varphi$ with strike direction normal to the *xz*-plane (described in Methods). Basement fault stability under loading is quantified by evaluating the $\Delta CFS$, which is defined as $\Delta CFS=\left|{\Delta \tau }_{s}\right|+\mu \left(\Delta {\tau }_{n}-\Delta p\right)$, where ${\Delta \tau }_{s}$ is the change in the fault shear traction, $\Delta {\tau }_{n}$ is the change in the total normal traction (positive in compression), $\Delta p$ is the pore pressure change, and $\mu$ is the coefficient of fault friction. Therefore, the $\Delta CFS \mathrm {\;is\;a\;function\; of}f\left({N}_{0}, {\theta }_{1}, {\theta }_{2}, a, \varphi ,\mu \right).$ Considering the dip of the basement faults in the Aravalli Delhi fold belt region as $\varphi =$ 60°, the range of frictional coefficient as $\mu =$ 0.3–0.6, effective load width of urbanization as $a=$ 65(± 2) km, and our estimate of the weight of the urban areas as ${N}_{0}$, we computed $\Delta CFS$ on a vertical NW–SE profile with stresses resolved on the dipping basement faults (Fig. 4). It appears that the elastic response to the urbanization weight in the Delhi surrounding region exerts loading-induced stress of at least ~ 1–4 kPa at the hypocentral region of the earthquakes.

The critical threshold stress value for seismicity induced by the external loading on a fault system (i.e., tidal loading, hydrological loading, teleseismic event, rainfall, building load, etc.) is also essential to understand the modulation of seismicity. It has been observed that the critical triggering threshold value for tidal loading is about 0.15−0.3°^39^ and less than 2 kPa for rainfall-induced earthquakes^40^. Further, Kundu et al.^41^ suggested that the threshold stress for groundwater unloading-induced 2015 Gorkha earthquake in Nepal is about 0.05 − 0.15 kPa/year. Similarly, the critical stress value for seismic waves is 0.1−10 kPa^42,43^ and 0.1–1 kPa for hydrological load-induced non-volcanic tremor along the Cascadia subduction zone^44^. However, Ziv and Rubin^45^ has argued that there is no threshold for earthquake-triggering or modulation. In the present study, the estimated stress induced by the urbanization weight in the Delhi surrounding region is higher than the previously estimated critical threshold stress value of various exogenous forcings. Hence, we have suggested that the stress induced by the urbanization weight in the Delhi surrounding region is sufficient for decadal seismicity modulation in the Delhi surrounding region.

Further, a comparison of the stress drops associated with smaller magnitude earthquakes (e.g., stress drops of 0.25–2 MPa^46,47^ associated with magnitude range 1–3) and the stress change due to urbanization around the Delhi surrounding region implies that this anthropogenic loading process might have substantial potential to reduce the seismicity rate for longer periods by stabilizing thrust faults in the basement. However, it is difficult to comment on the exact time frame. We also examined the possibility of the Yamuna river-induced erosional process and its influence on the stress-triggering mechanism of the Aravalli Delhi fold belt hosted basement faults because erosion has been reported as a viable mechanism in the active thrust fault region of Taiwan^48^ and the classical plate interior region of New Madrid Seismic Zone^49^. It turns out that the contribution of erosion-induced stress change is insignificant in comparison to the anthropogenic urbanization-induced stress change in the Delhi surrounding region. Hence, we ruled out that possibility (described in Methods).

### Nonlinear effects from building loads: contribution from primary and secondary settlement

It can be noted that the Delhi surrounding region has been covered by alluvium cover with reported thickness up to 300 m along with ~ 10–12 m thick soil layers in the immediate vicinity, which is occasionally traversed by the linearly elongated quartzite ridges that act as a basement rock of the area^34,35,50^. Hence, surface rock and soil cover in the Delhi and surrounding vicinity exhibit time-dependent nonlinear deformation characteristics, e.g., initial and secondary compaction of near‐surface soils; compression‐induced void closure and dewatering process; and long‐term creep and compaction in the post-construction phase^51–53^. As a result, near‐surface rock and soil skeletons under loading-induced stress experience two stages of nonlinear settlement: primary and secondary settlements^51^. Elastic compression and pore-fluid drainage from the soil cover is considered as the primary settlement, whereas longer‐term creep and compaction mechanism that may continue indefinitely has been considered as the secondary settlement^53^.

**Figure 5:**
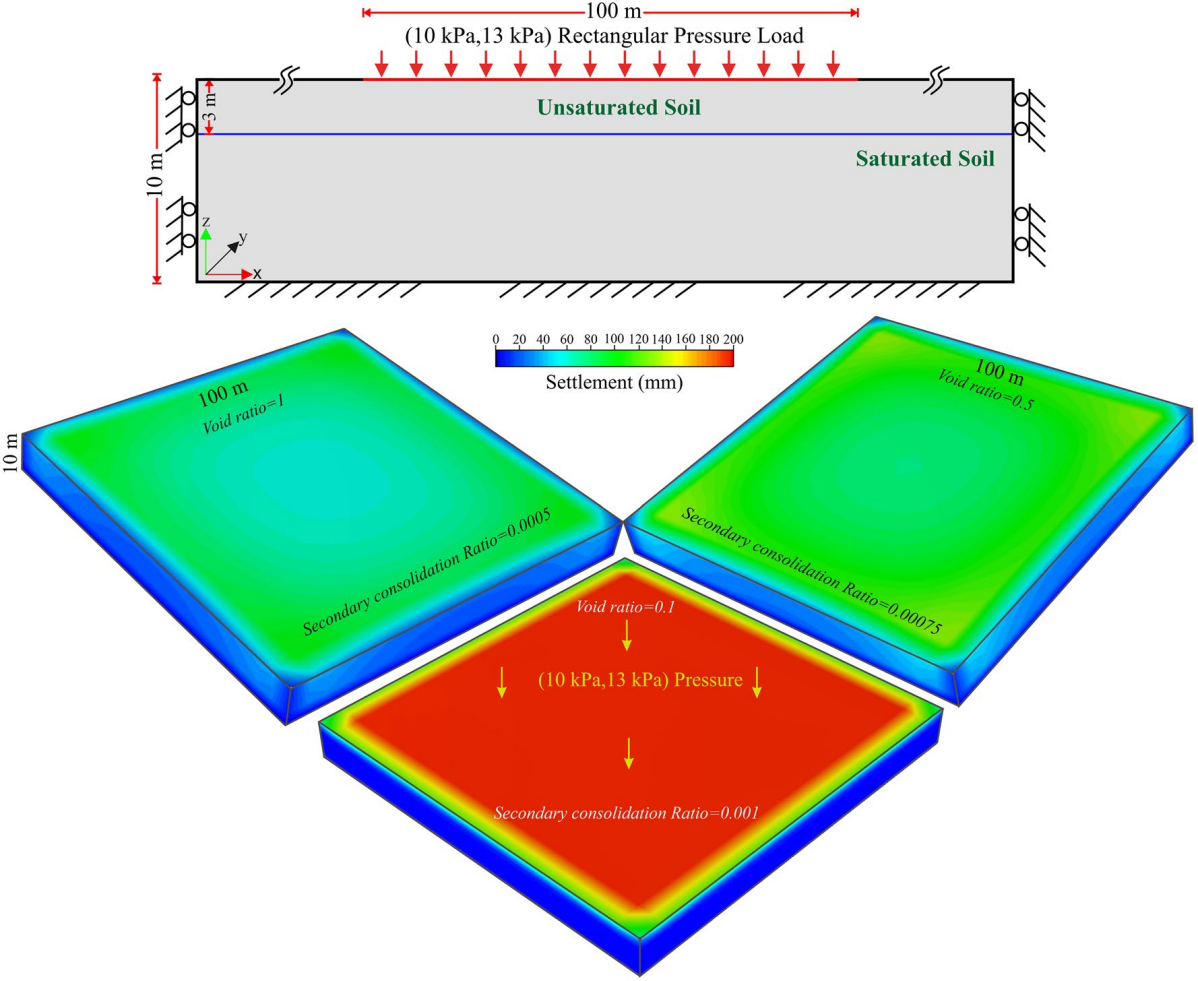
Total settlement analysis of the model. Total settlement induced by 10 kPa and 13 kPa total surface load for 100 years of simulation considering three different void ratio values (i.e., 1, 0.5, 0.1) and three different Secondary Consolidation Ratio values (0.0005, 0.0075, 0.001) arranged as left, right, and center figures, respectively. This figure was generated using Settle 3D (version 2.0 URL: https://www.rocscience.com/software/settle3).

**Figure 6:**
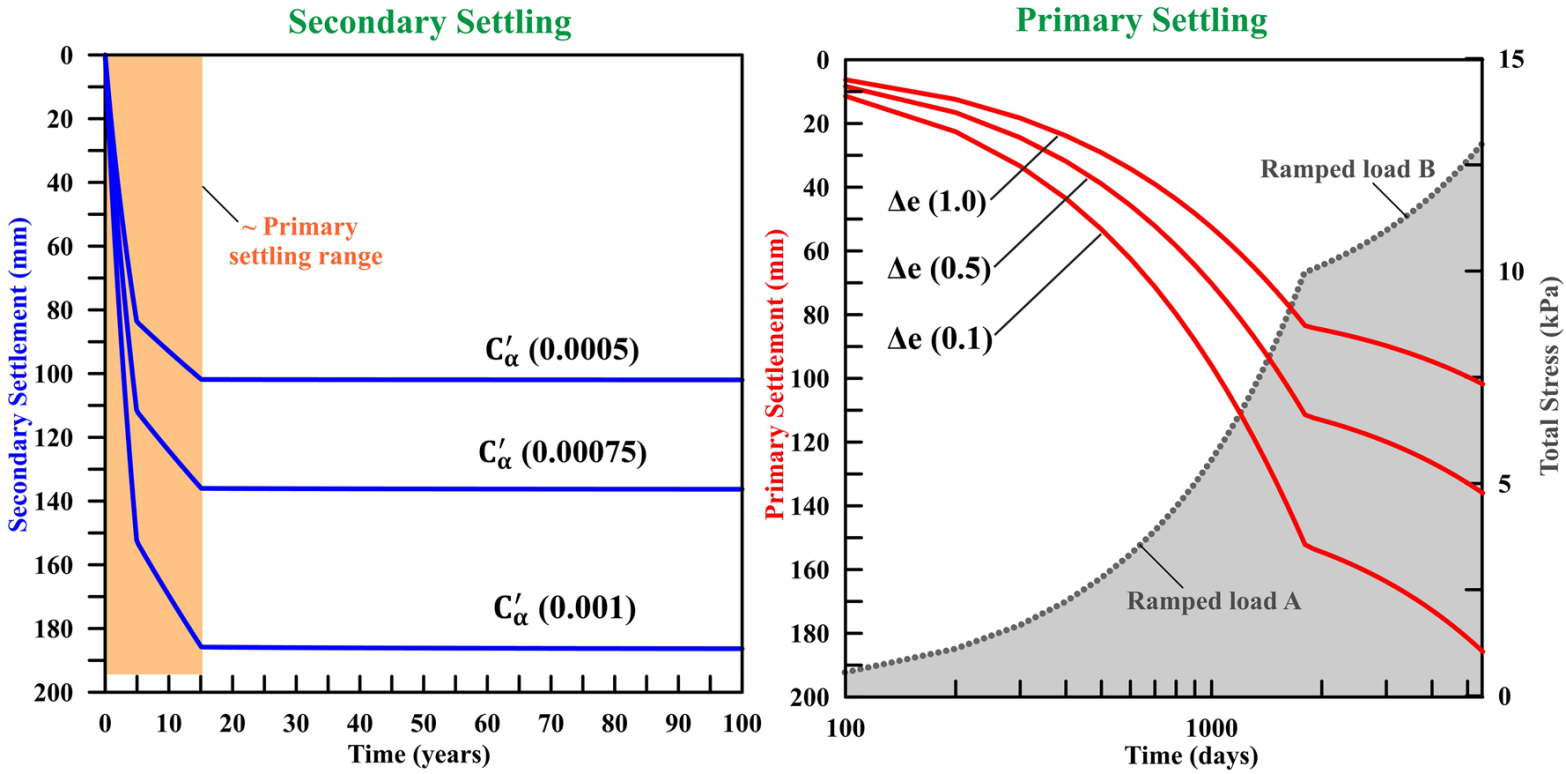
Primary and secondary settlement variation with time. *Right*: The ramped loads A and B are applied to compute the impact of varying urbanization load on elastic soil. Soil compaction, void closure, and dewatering leads to primary settlement from building load, which has been plotted here for three void ratio values over a 15-year period. *Left*: Combined impact of the long-term creep process, primary settlement for different void ratios, and secondary consolidation-induced settlement computed for 100 years. This figure was generated using Grapher graphical application (version 16.6.478 URL: https://www.goldensoftware.com/products/grapher).

To quantify primary settlement and associated time-dependent nonlinear deformation under the building load, we present a simplistic yet realistic model that accounts for the rapid urbanization in the Delhi region (Fig. 1b). The finite element simulation model uses the Settle3D code (https://www.rocscience.com/software/settle3) (described in Methods, and Figure S2). The effect of secondary settlement has been computed using the expression from Buisman's^54^ equation: ${S}_{c}={{C}_{a}{\prime}H}_{c}\mathrm{log}\left[\frac{{t}_{2}}{{t}_{1}}\right]$, where secondary settlement is a function of the coefficient of secondary compression (${C}_{a}{\prime}$), effective soil thickness (${H}_{c}$), void ratio of soil at the end of primary settlement (${e}_{p}$), and start and end times (${t}_{1} , {t}_{2}$) (described in Methods). We implemented two rectangular (100 m × 100 m) ramp loads, A and B, of magnitudes ~ 10 kPa and 13 kPa, respectively, applied over the soil surface at different times using standard properties of soil in the Delhi surrounding region (Figure S2, Table 1). Detailed model geometry and boundary conditions are presented in the supporting documents and in Figs. 5 (described in Methods). The effects of the two phases of the primary settlement are simulated for 15 years, and the total settlement is calculated by including the secondary settlement for ~ 100 years. It has been observed that the primary settlement mostly occurs due to void closure and subsidence caused by the building load within a few years of construction. The simulated total settlement for the loading area has been presented in Fig. 6 assuming a specific void ratio range (Δe) and coefficient of secondary compression (${C}_{a}{\prime}$). The loading condition, primary settlement, and total settlement with time have been presented at the model centre (Fig. 6). As per the model, the total settlement for ~ 100 years is ~ 160–190 mm, which includes the effect of dual phases of primary settlement and the secondary settlement. The primary settlement rate over the past ~ 15 years is calculated to be ~ 3–10 mm/year, which is comparable with the surface subsidence rate of ~ 2–18 mm/year observed in the radar data around the Delhi surrounding region. Although the geology is extremely localized, soil characteristics and the heterogeneous nature of groundwater abstraction are also contributing to the spatiotemporal discrepancy between the calculated and observed subsidence rates, which is difficult to account for.

### Linking Delhi urbanization to long-term seismicity modulation

**Figure 7:**
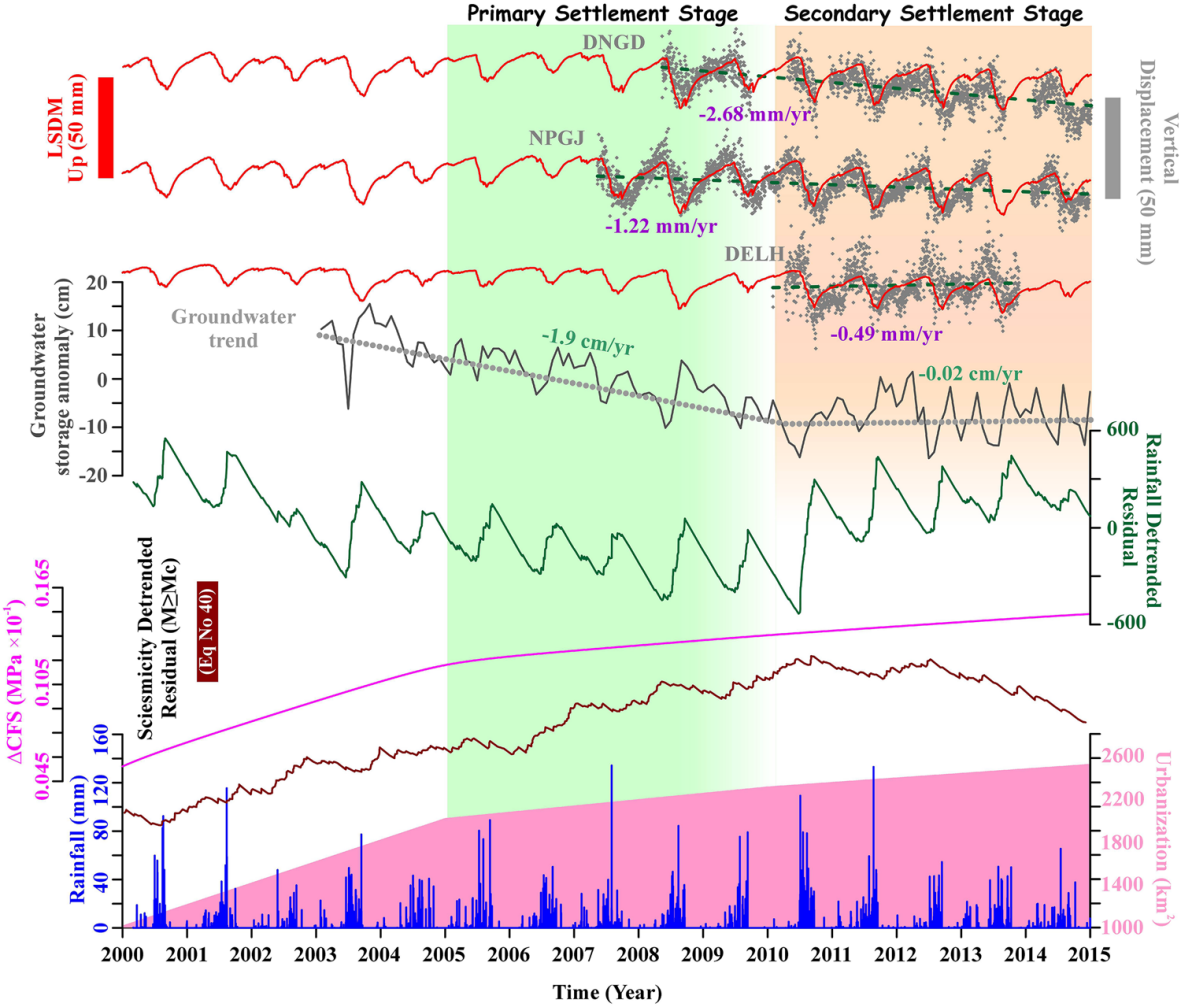
Correlation between vertical displacement of LSDM and GPS, Groundwater storage anomaly, seismicity, and rainfall. Time series representation of vertical displacement from LSDM and GPS at three sites DNGD, NPGJ, and DELH, Groundwater storage anomaly, which are derived from the GRACE and GLDAS observations in the study region. Take note of the groundwater level's declining trend (1.9 cm/year) from 2002 to 2009, followed by a period of stability. The cumulative change in the Aravalli Delhi fold belt associated seismicity, CFS, and regional rainfall are presented in the lower panels. Slope shifts in detrended cumulative seismicity, groundwater storage anomaly, CFS, and detrended cumulative regional rainfall occur at the same time (marked by the orange shading). The pink-shaded region represents cumulative area change from 2000 to 2015, which shows a rapid urbanization trend in Delhi and its surrounding region from 2000 to 2005; afterward, the trend became consistent. Note a rapid increase in primary urbanization during the period of 2005–2009 (marked by the green shading). The orange shading represents a relatively stable trend during 2010–2015. This figure was generated using Grapher graphical application (version 16.6.478 URL: https://www.goldensoftware.com/products/grapher).

To represent decadal seismicity modulation around Aravalli Delhi fold belt and the Delhi surrounding region, we generated an aftershock-depleted relocated catalogue (2000–2020) and estimated the lower bound of the magnitude threshold in the catalogue (Mc = 2.5) that remains stable over time during the observation period (described in Methods). Figure 7 represents a composite time series for the period of 2000–2015 to compare (a) groundwater storage anomaly derived from the GRACE and GLDAS observation; (b) observed surface deformation along with hydrological mass variation captured by cGPS displacements and satellite-based hydrological model prediction by LSDM, (c) detrended cumulative change in the corresponding seismicity rates for declustered catalog (M ≥ Mc), (d) regional seasonal rainfall and its detrended-residual pattern, (e) $\Delta CFS$ induced by urbanization load (considering *µ* = 0.6) and (f) effect of Delhi urbanization and the primary settlement rate. The visual inspections of these composite time series exhibit good correlations with the cumulative change in the decadal seismicity rate around the Aravalli Delhi fold belt. It can be noted that there is a significant drop in the seismicity rate from 2010 onwards, with an initial steady rise in the seismicity rate from 2000. This decadal change in the seismicity rate can be connected to two mechanisms, the decadal change in the groundwater storage anomaly and the primary settlement induced by building loads, both of which modulate the stability of basement thrust faults. The rapid decline in groundwater storage change (~ 1.9 ± 0.6 cm/year) during the period of 2003–2009 (Fig. 7), followed by a relatively stable trend (0.02 ± 0.5 cm/year) during 2010–2015 can be linked with the basement fault stability due to combined aquifer contraction and basement rock expansion due to groundwater withdrawal. These mechanisms act together to modulate the effective stress regime and the seismicity rate on the basement faults^36^. Groundwater storage change is also converging with a rapid decline in the regional rainfall during 2003–2009, followed by significant recharge of the aquifer in the later period (2010–2015) (Fig. 7). Regarding the second mechanism, the addition of a significant amount of vertical stress on the ground during the rapid urbanization process, along with time-dependent nonlinear effects from the building load, is capable of modulating the subsurface state-of-stress by stabilizing basement thrust faults and reducing the seismicity rate (Fig. 7, Figure S3). Hence, both mechanisms, i.e., groundwater storage change and urbanization, are complimenting each other, and both appear to be significant for decadal seismicity modulation around the Aravalli Delhi fold belt. However, it is difficult to discriminate their relative contributions to the basement fault stability. Moreover, the exact time frame of interaction between basement fault stabilization and Delhi urbanization awaits further investigations because it is difficult to comment on the temporal extent of urbanization-induced stress that acted to reduce the seismicity rate in the basement faults. Further, we acknowledge that the seismicity rate of the Delhi region also exhibits significant seasonal variation in annual scale; the seismicity level is relatively low during the time period of seasonal hydrological loading (Figure S4).

## Concluding remarks

**Figure 8:**
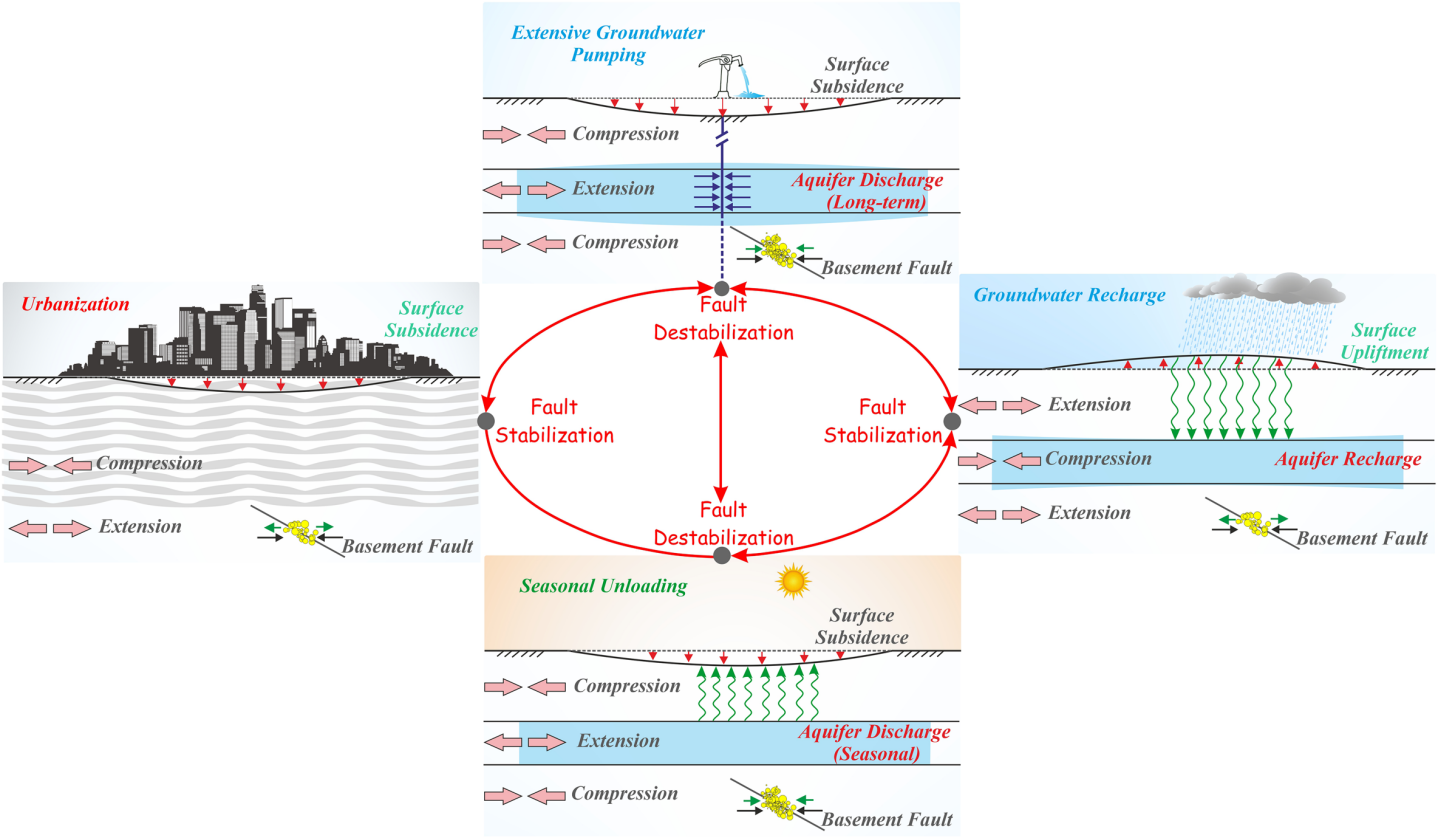
Schematic representation of crustal loading and unloading, ground subsidence, and faulting associated with groundwater extraction, aquifer recharge, seasonal unloading, and urbanization. The light blue color region indicates the unconfined aquifer. Compression of basement rocks near locations of extensive pumping favors reverse faulting. Crustal unloading due to groundwater extraction and seasonal unloading causes basement expansion, which can apply horizontal compression (green arrows) on bounding faults that are far from the aquifer. This compression adds to the long-term secular contraction (black arrows). Aquifer recharge and urbanization processes cause crustal loading, which applies horizontal extension (left and right panel) on the aquifer-bounding faults. This figure was generated using Corel Draw graphical application (version 22.2.0.532 URL: https://www.coreldraw.com/en).

Unlike other perceptible environmental changes caused by urbanization, changes in the subsurface are more difficult to perceive and observe. However, the impacts of subsurface change are not confined to the urbanized region and extend to regions beyond. To test the hypothesis that rapid urbanization is disturbing the subsurface state-of-stress, characteristics of a mega-urbanized region surrounding Delhi were investigated. Based on the above analysis, modeling results of the weight of the city and urbanization, and related discussion, it can be inferred that the decadal variation of seismicity rate in the Aravalli Delhi fold belt and the surrounding region has possibly been influenced by the rapid urbanization process and time-dependent nonlinear effects of the building load along with non-tectonic anthropogenic groundwater pumping. Further, the seismicity rate at the annual scale also reflects seasonal variation induced by the hydrological loading process. Although the mechanism of faulting is a tectonically controlled process and the precise rate of tectonic stress accumulation is unknown due to a lack of geophysical constraints in the Aravalli Delhi fold belt plate interior region, we argue that the ongoing tectonic loading during the inter-seismic phase of the earthquake cycle of the basement faults in the Aravalli Delhi fold belt experienced significant horizontal compression (and extension) depending upon the non-tectonic loading (and unloading) process under a diverse set of scenarios. This has significantly influenced the secular interseismic compression at seismogenic depth over the past several decades (Fig. 8). It can be argued that a significant component of non-tectonic horizontal compression is added to the secular interseismic compression from the regional tectonic load due to extensive pumping of groundwater over a decadal time scale and seasonal unloading over a yearly time scale. The resulting net increase in horizontal compression promotes fault destabilization. However, rapid urbanization and time-dependent nonlinear effects of building loads add a significant component of non-tectonic horizontal extension to the secular interseismic compression, thereby reducing the resultant secular interseismic compression and stabilizing the basement faults in the Aravalli Delhi fold belt. The aquifer recharge as part of the urbanization process is also causing the same influence (Fig. 8).

In the context of the present study, Parsons^4^ calculated the subsurface state-of-stress induced by the weight of urbanization for a developed region like San Francisco, USA. However, it did not relate the weight of urbanization to decadal seismicity modulation. Similarly, Lin^25^ discussed the load of a single building, the Taipei 101 tower, on normal faults and associated seismicity in the Taipei basin, Taiwan. It reported that the seismic energy and the number of micro-earthquakes began to increase slightly during the construction of the 508 m tall building above the Taipei basin and sharply increased after the completion of tower construction. Hence, we consider the present study to be new and probably the first report discussing the direct effect of urbanization on crustal deformation and associated seismicity modulation. We acknowledge that it is difficult to comment on the relative contributions of non-tectonic stresses on the basement fault, but still, it can be argued that for developing countries like India, urban land expansion and associated “weight of the city” appear to be new focal points for understanding the subsurface state-of-stress and seismicity modulation in the region.

## Material and methods

### Land use classification scheme of the Delhi urbanization area

The focus of the study is the capital city of India, New Delhi, and its surroundings, with the geographical extent of 28°–29.30° N and 76°–78° E (Fig. 1a). From data pre-processing to LUC accuracy assessment, our analysis used a cloud-based computing platform–Google Earth Engine (GEE)–and our local computing system. At unprecedented scales, GEE parallelizes screen map area to allow intensive cloud computational processes such as machine learning (ML)-based LUC^62,63^. By adopting the GEE-ML algorithm, the region was mapped with ‘Level I’ primary dominant LUC classes at 30 m resolution, employing Landsat TM, ETM + and OLI, at 5-year intervals since 1990. The choice of this classification level was backed by the imposition of spectral signature constraints across different Landsat platforms^63,64^. The hierarchical LUC of the region was defined by five broad land-cover classes: barren (LUC differ spectrally from the dominant classes and the class includes barren land, permanent fallow, wetlands, etc.), built-up (artificial and unnatural structures in urban centres and their buffer areas), cropland (irrigated or rainfed cropping area including fallow and permanent), forest (includes vegetation dominated areas with trees over 2 m in height; includes closed, open, mixed, sparse and scattered land types with ≥ 20% canopy cover), and water (perennial inland water bodies). To rationalize the classification system for geomechanical analysis, some of the sub-classes were grouped together. For example, grassland and shrubland were merged into the forest class, and non-perennial water bodies were merged into the barren class. Despite its high spatial resolution, the LUC frame utilized a combination of high-resolution toposheets and spatially explicit secondary census information for its robust resolution and spatial consistency.

### Pre-processing of primary data and associated auxiliary variables

To retain the focus on the LUC, our study trimmed the spectral effects raised, such as redacting the scattering and absorption effects, sensor mismatch, footprint and acquisition mismatch, etc., during pre-processing^62,64,65^. Therefore, due to the highest data quality assurance, our analysis employed only the surface reflectance data from the Tier-1 Landsat scenes, which were atmospherically corrected and topographically, radio-metrically and geometrically calibrated. We matched our analysis with the established Landsat data processing framework, which has been reflected by many studies. The calibrated datasets were harmonized by masking saturated pixels, clouds and cloud-shadows to maintain the spectral similarity across multiple Landsat sensors. Despite the scan line corrector failure for the Landsat ETM + images, our analysis rectified the scenes for the year 2005 with the ‘USGS SLC-off gap-filling algorithm’ to retain the reliable consistency of the time series comparison^62,65^. To minimize the bias in the analysis, the data pixels were excluded from the analysis if it did not fit in the quality flags. However, studies reported that, 99% of the data products from the GEE platform are more consistent than the conventional data platforms. At each analysis epoch (i.e., 1990, 1995, 2000, 2005, 2010, 2015 and 2020), the spectral bands were stacked, and the median values were computed at each pixel. Apart from the orthodox band composites (i.e., Blue, Green, Red, NIR, SWIR 1 and 2), auxiliary spectral indices derived from the parent data bands, namely normalized difference built-up index (NDBI)^66^, normalized difference vegetation index (NDVI)^62^, enhanced vegetation index (EVI)^67^, soil-adjusted vegetation index (SAVI)^68^, normalized difference water index (NDWI)^69^ and land surface temperature (LST)^70^, were stacked with the composites to improve the classification accuracy.

### Supervised classification scheme

We use the random forest (RF) land cover classifier method^62,64,71^ which is one of the most reliable algorithms hitherto and is widely used among researchers for its processing speed, noise handling, and higher accuracy. The human-interpreted semi-automated random sampling process was employed for constructing the training and validation datasets. Being the pseudo-ground truth data, we corroborated that the sample was stratified to the maximum throughout the scene. The sampled spectral signatures were labelled using the administrative maps of the survey of India toposheets. Further, to eliminate the dubious samples and potential outliers, non-supervised classified spectral signatures were overlaid on the pseudo-ground truth data^62^. We excluded the ordinated samples to potentially improve the classification accuracy. To substantiate a homogenous spatial coverage, a final visual correction was employed by removing the outliers. The final decider sample spectral signatures were 15,834, with 11,251 and 4583 being the number of training and validation samples. Since spatial autocorrelation is a menace to the validation scheme, if the validation signatures are in close proximity to the training signatures, we corroborate the truancy of training samples away from the validation samples, at a minimum of 100 m buffer. Further, for explicit validation, the Landsat scenes were partitioned using a hexagonal scheme as proposed by Gong et al.^72^ by proportioning the sample signatures.

### Accuracy assessment and land-cover change analysis

Being the most prominent accuracy assessment method among the researchers, confusion matrices (with reports of overall accuracy, user accuracy, and producer accuracy) were developed by comparing the accuracy of multiple LUC. Readers are directed to the extensive documentation on the confusion matrix for accuracy assessment by Congalton^73^. Using the validation samples, different accuracy metrics were calculated for each epoch. As proposed by Foody^74^, confusion matrices depict the results by pretermitting the small accuracy differences. The kappa coefficient (κ) was also used in assessing the accuracy of LUC maps for statistical stability. This is relevant, especially when multiple sample spectral signatures are used as classifiers, which is not our case. With the theme being urban land expansion, a single post-classification process was applied to minimize the temporal bias, where the pixels other than the ‘built-up area’ were reclassified and excluded for a robust temporal transition and to superimpose it over the subsurface stress regions. However, the classification accuracy and the LCC transitions were calculated prior to the post-classification. Each validation signature of the ‘built-up’ class was visually inspected with Google Earth to track the temporal dynamics of the area change. To intercept the bias in the ‘built-up’ area change, the error-adjusted area, as proposed by Olofsson et al.^75^, was employed with ρ > 0.05. The error margin and the confidence intervals in the land cover change were calculated using a normal distribution function as proposed by Schmidt and McCullum^76^:

1 $$\sigma =\sqrt{\sum_{i=1}^{n}\frac{{W}_{i}\times {\widehat{p}}_{ij}-{{\widehat{p}}_{ij}}^{2}}{{n}_{i}-1}}$$

where σ is the standard error, ${W}_{i}$ is the stratum weight, ${\widehat{p}}_{ij}$ is the pixel-based error matrix and ${n}_{i}$ is the number of total classified points, respectively. The total number of classes is represented by $n$ and $j$ represents the area-based error index of various classes.

### Groundwater storage change computation

The Water Balance Method based annual groundwater storage change has been estimated using more than 15 years of Gravity Recovery and Earth Climate Experiment (GRACE) and global land data assimilation system (GLADS) water content data^77–81^. GRACE RL05 data of $(1^\circ \times 1^\circ )$ spatial resolution used for computation is provided by the National Aeronautics and Space Administrations (NASA) based on spherical harmonics solutions of Jet Propulsion Laboratory (JPL) (https://grace.jpl.nasa.gov/). We used NASA GLDAS’ four vertical levels of monthly soil moisture data with a spatial resolution of $\left(1^\circ \times 1^\circ \right),$ available at https://disc.gsfc.nasa.gov/datasets?keywords=GLDAS, in order to obtain the primary land surface flux and storage component for the same time period as the GRACE data. The groundwater storage change of the total terrestrial water storage can be estimated from the GRACE data. This has been expressed as:

2 $$\Delta {\text{GW}}=\Delta S-\Delta SWE-\Delta SW-\Delta SM$$

where $\Delta$ GW is the groundwater storage change. $\Delta$ S, $\Delta$ SWE, $\Delta$ SW, and $\Delta$ SM represent the change in total terrestrial water storage, snow water equivalent, surface water storage, and soil moisture, respectively. The anomalies of changes in the snow water equivalent, surface water storage, and soil moisture are extracted from the NASA Noah land surface model. Monthly time-series of $\Delta$ GW are used to determine the statistical significance of the trend by employing the non-parametric Mann–Kendall test^82,83^. Presented results are significant at the 95% confidence level (*p* < 0.05). Further, based on the respective errors of the specific components, the trend error in groundwater change is estimated using the expression below:

3 $${\sigma }_{GW}=\sqrt{{({\sigma }_{S})}^{2}+{({\sigma }_{SWE})}^{2}+{({\sigma }_{SW})}^{2}+{({\sigma }_{SM})}^{2}}$$

where $\sigma$ represents the one-sigma trend error in the component mentioned in the subscript.

### Ground subsidence from multi-sensor Radar data

Globally active deformation of ground in populous regions is frequently estimated using the interferometric synthetic aperture radar (InSAR) measurements^84–86^. For the detection of surface deformation in wide regions with high accuracy and mm level precision, generally, SAR sensors are preferred in comparison to any other technique^84,87–89^. For estimation of surface subsidence over the Delhi region, 45 Cosmo–Skymed images are processed, which were acquired between June 8, 2011 and November 15, 2017. We have adopted the differential InSAR technique to quantify the ground displacement in the line-of-sight (LOS) direction of the satellite, constraining the phase difference of temporally separated SAR images. Using this technique, the land subsidence over the Delhi and adjacent region has been mapped with high precision and is presented in Fig. 3c.

### Building load-induced elastic stress model

The effect of computed mass over the elastic crust and fault stability has been quantified using the *ΔCFS*. For that, the induced stress from the vertical line load (${N}_{0}$ in N/m) at any point (*P*) within a homogenous elastic half-space at a specific depth is estimated. The three components of the 2-D stress tensor (${\tau }_{xx}$, ${\tau }_{zz}$, ${\tau }_{xz}$) are represented as functions of the geometrical position of the load^38^,

4 $$\left.\begin{array}{c}{\tau }_{xx}= \frac{{N}_{0}}{\pi a}\left[\left({\theta }_{1}-{\theta }_{2}\right)+\mathrm{sin}\left({\theta }_{1}-{\theta }_{2}\right)\mathrm{cos}\left({\theta }_{1}+{\theta }_{2}\right)\right]\\ {\tau }_{zz}= \frac{{N}_{0}}{\pi a}\left[\left({\theta }_{1}-{\theta }_{2}\right)-\mathrm{sin}\left({\theta }_{1}-{\theta }_{2}\right)\mathrm{cos}\left({\theta }_{1}+{\theta }_{2}\right)\right]\\ {\tau }_{xz}= \frac{{N}_{0}}{\pi a}\left[\mathrm{sin}\left({\theta }_{1}-{\theta }_{2}\right)\mathrm{sin}\left({\theta }_{1}+{\theta }_{2}\right)\right]\end{array}\right\}$$

where ${\theta }_{1}$ and ${\theta }_{2}$ are the angles from both edges of the line load, measured clockwise from the positive *x* direction, *z* is positive downward, and $a$ is the width of the load. The shear ${(\tau }_{s}$) and normal stress ${(\tau }_{n}$) components are resolved on a receiver fault plane, dipping at an angle of $\phi$ with the strike direction normal to the xz-plane:

5 $$\left.\begin{array}{c}{\tau }_{s}={\tau }_{zz}{\mathrm{cos}}^{2}\left(\phi \right)-2{\tau }_{zx}\mathrm{sin}\left(\phi \right)\mathrm{cos}\left(\phi \right)+{\tau }_{xx}{\mathrm{sin}}^{2}\left(\phi \right)\\ {\tau }_{n}={(\tau }_{zz}-{\tau }_{xx})\mathrm{sin}\left(\phi \right)\mathrm{cos}\left(\phi \right)+{\tau }_{xz}{(\mathrm{cos}}^{2}\left(\phi \right)-{\mathrm{sin}}^{2}\left(\phi \right))\end{array}\right\}$$

The $\Delta CFS$ is given by:

6 $$\Delta CFS=\left|{\Delta \tau }_{s}\right|+\mu \left(\Delta {\tau }_{n}-\Delta p\right)$$

where $\mu$ is the fault friction coefficient and $\Delta p$ is the pore-fluid pressure change.

### Building load-induced nonlinear effects from primary and secondary settlement

The Boussinesq equation is the most commonly used expression to obtain the stress distribution induced by a vertical load, assuming the soil is an elastic, isotropic, and homogeneous medium. The Settle 3D FEM model^60^ uses the closed-form expressions of the stress profiles at the corner of a uniformly loaded rectangular area and, hence, provides more robust results. We used this FEM modeling approach to estimate the total settlement due to the building load. The total settlement is the sum of three components Immediate Settlement (Initial), Settlement due to consolidation (Primary Settlement), and Secondary Settlement (Long Term Creep).

**Table 1:** Assumed properties of soils in the Delhi and surrounding regions for modeling the building load-induced nonlinear effects from primary and secondary settlements.

Soil properties	Symbol	Value considered	Range considered	Remarks/source
Unsaturated unit weight	$\gamma$	18 kN/m^3^	15–22 kN/m^3^	Delhi Silty Sand^55–57^
Saturated unit weight	$\gamma {\prime}$	21 kN/m^3^	18–24 kN/m^3^	
Poisson ratio	$\upsilon$	0.3	0.2–0.45	
Compression Index	${C}_{c}$	2.65	1.5–4	Standard for Silty Sand^57–59^
Recompression Index	${C}_{r}$	0.26		1/10th of compression Index
Pre-consolidation stress	${P}_{c}$	100 kPa	60–160 kPa	Standard Atmospheric Pressure^60,57,58^
Initial void ratio	${e}_{o}$	0.1–1	–	Variable^4^
Hydraulic conductivity	$k$	0.00584 m/year	0.00221–0.00947 m/year	Typical values for SiltySand^57,59,61^
Skempton’s coefficient	$B$	0.98	0.94–1	Delhi Silty Sand^56^
Secondary compression Index	$C_{a} /C_{r}$	0.0005–0.001	–	Variable^4^

The Immediate Settlement is an instantaneous effect due to the applied load and is assumed as linear elastic. Settle3D computes the strain of each element using 1D elastic modulus and effective stress given as:

7 $$\left.\begin{array}{c}E={E}_{o}\frac{\left(1+v\right)\left(1-2v\right)}{1-v}\\ \epsilon =\frac{\Delta \sigma }{{E}_{o}}\end{array}\right\}$$

where $v$ is Poisson’s ratio and ${E}_{o}$ is the initial elastic 1D modulus (constrained modulus), $\epsilon$ is vertical strain, and $\Delta \sigma$ is the change in the total vertical stress. The bottom surface is assumed to be fixed, and the displacement of the element above the bottom is given by:

8 $$\delta =\Delta z=\epsilon {H}_{c}$$

where ${H}_{c}$ is the thickness of the soil layer. The settlement of the ${i}^{th}$ element is then given by the sum of settlement of the element below $(i+1)$ and the settlement of the sublayer element:

9 $${\delta }_{i}={\delta }_{i+1}+{\epsilon }_{i}{{H}_{c}}_{i}$$

Primary settlement progresses gradually by closure of the void spaces and increase in the effective stress. The non-linear material modulus is a function of stress. The ratio of the volume of voids to the volume of solids is the void ratio $e={V}_{v}/{V}_{s}$. The preconsolidation stress ${P}_{c}$ is the maximum effective stress a soil layer experienced in the past. The void ratio and the logarithm of the effective stress relation is given by recompression index ${C}_{r}$. For the primary settlement, the change in void ratio $\Delta e$ can be calculated from the initial effective stress ${\sigma }_{o}$ and the final effective stress ${\sigma }_{f}$ as follows:

10 $$\Delta e=-{C}_{r}log\left(\frac{{\sigma }_{f}}{{\sigma }_{o}}\right)$$

The relation between the vertical strain and the void ratio is given by

11 $$\epsilon =-\frac{\Delta e}{{1+e}_{o}}$$

where ${e}_{o}$ is the initial void ratio. Then the change in strain is given by:

12 $$\Delta \epsilon =\frac{{C}_{r}}{{1+e}_{o}}log\left(\frac{{\sigma }_{f}}{{\sigma }_{o}}\right)$$

The secondary settlement, or creep, occurs at constant effective stress for some types of soils. Buildings are usually constructed on soils and fractured rock. During the time of construction, and till the completion of the initial compaction period after construction, settlement of the soil occurs. This time-varying behavior of soil under stress results from its compositional and constitutive properties^52^.

Further, the secondary settlement of soil^54^ is given by:

13 $${S}_{c}={{C}_{a}{\prime}H}_{c}\mathrm{log}\frac{{t}_{2}}{{t}_{1}}$$

where the coefficient of secondary compression ${C}_{a}{\prime}$ is:

14 $${C}_{a}{\prime}=\frac{{C}_{a}}{1+{e}_{p}}$$

where ${t}_{1}\mathrm{and} {t}_{2}$ are the start and end times of settlement, ${e}_{p}$ is the void ratio at the end of primary settlement, and ${C}_{a}$^90^ is expressed as:

15 $$C_{a} = \frac{\Delta e}{{\log \left( {t_{2} /t_{1} } \right)}}$$

### Erosion due to River Yamuna and its effects on subsurface stress

The river Yamuna originates from the Yamunotri Glacier at an altitude of 6330 m above the mean sea level (msl) in the Mussorie range. After passing through the Siwalik hills, it travels to its confluence with the Ganges at Allahabad (100 m above msl) from Tajewala (370 m above msl). Jha et al.^91^ estimated the rate of erosion in the upstream region of Yamuna averages about 2178 t/km^2^/year at Baghpat and decreases downstream to 308 t/km^2^/year near the confluence with the Ganga at Allahabad. The width of Yamuna in Delhi varies at several locations as Gurusamy and Jayaraman^92^ estimated it at Yamuna barrage (Delhi) 255.9 m, Railway bridge 193.8 m, Nizamuddin bridge 309.6, and Noida toll bridge 418.3 m. To estimate the influence of sediment erosion by Yamuna River on the subsurface stress modulation around the Delhi surrounding region, we computed the $\Delta CFS$ using the above method, where the erosion rate of 2178 t/km^2^/year has been converted to unloading of a line load and resolved over the 60° dipping basement fault using two values of the frictional coefficient: 0.3 and 0.6. The estimated $\Delta CFS$ is on the order of 10^−5^ Pa, which is insignificant in comparison to the anthropogenic urbanization-induced stress change in the Delhi surrounding region.

### Hydrological loading models predicted vertical displacements at cGPS sites

In order to estimate the surface deformation caused by seasonal hydrological mass variation at cGPS site coordinates, we consider the elastic loading models from both ground and satellite-based hydrological analysis, e.g., Land Surface Discharge Model (LSDM). We have considered the LSDM hydrological model provided by the GFZ (http://rz-vm115.gfz-potsdam.de:8080/repository). The hydrological load is taken from the hydrological LSDM version v1.3, which includes 24-hourly estimates of soil moisture, snow and surface water mass in rivers and lakes on a regular grid of 0.5° ×  0.5°^93^. Hydrologically induced elastic surface deformation is calculated by convolving Farrell’s Green’s function with the modeled hydrological mass distributions from the LSDM. The elastic deformation has been computed in the centre of Earth’s frame (cF), which closely follows deformation on seasonal and shorter timescales^94^ and on the basis of load Love numbers given for the elastic Earth structure model “ak135”^95^. All the cGPS time series from the Delhi surrounding region can be obtained from http://geodesy.unr.edu/NGLStationPages/gpsnetmap/GPSNetMap.html. Precipitation data used in this paper and used in Fig. 7 can be obtained from the Global Precipitation Climatological Centre (GPCC, http://www.esrl.noaa.gov/psd/).

### Delhi seismicity catalog and catalog completeness

To make these observations more robust, we generated an aftershock-depleted relocated earthquake catalog from 2000 to 2020, controlled by the National Center of Seismology (NCS), Delhi, using the declustering approach^96^. To establish the catalog completeness (Mc), we analyzed the Gutenberg-Richter relation of the raw catalog of the region during the study period (2000–2020) using a maximum likelihood approach^97^. We find that the lower magnitude threshold (Mc = 2.5) in the catalog remains stable over time during the observation period (Figure S5). The earthquake catalog data can be downloaded from NCS, available at https://seismo.gov.in.

## Supplementary Information

Supplementary Figures.

## Data Availability

All the data and computational analysis used in this present study are presented in the text and supporting documents. Satellite datasets employed in this study are freely accessible from the Google Earth Engine cloud platform under the open data policy adopted by the Landsat program of the U.S. Geological Survey; administrative maps of the survey of India toposheets (1:50,000) are freely accessible from https://soinakshe.uk.gov.in/. Precipitation data used in this paper can be archived at Global Precipitation Climatological Centre (GPCC, http://www.esrl.noaa.gov/psd/). GPS time series from the Delhi surrounding region can be archived from http://geodesy.unr.edu/NGLStationPages/gpsnetmap/GPSNetMap.html. We used Settel3D FEM simulator (https://www.rocscience.com/) for settlement analysis. The earthquake data from NCS are available at https://seismo.gov.in/. All the other relevant data are available from the corresponding author upon reasonable request.
