# Supplementary material for: Delhi urbanization footprint and its effect on the earth’s subsurface state-of-stress through decadal seismicity modulation
Source: Sci Rep. 2023 Aug 3;13:11750. doi: 10.1038/s41598-023-38348-7 (PMC10400649; doi:10.1038/s41598-023-38348-7)
Supplement: Supplementary file 1 — Supplementary Figures. [file 41598_2023_38348_MOESM1_ESM.docx]

**Supporting documents**

**Delhi urbanization footprint and its effect on the earth’s subsurface state-of-stress through decadal seismicity modulation**

**Deepak K. Tiwari^1^, Manoj Hari^1,2^, Bhaskar Kundu^1*^, Birendra Jha^3^, Bhishma Tyagi^1^, Kapil Malik^4^**

**^1^**Department of Earth and Atmospheric Sciences, NIT Rourkela, Rourkela-769008, India

**^2^**Terrestrial Sciences Section, Climate and Global Dynamics, National Center for Atmospheric Research, Boulder, 80307, USA

**^3^**Department of Chemical Engineering and Materials Science, University of Southern California, Los Angeles, CA 90007‑1211, USA

**^4^**Indian Institute of Technology (ISM), Dhanbad 826004, India

***Corresponding author:** Bhaskar Kundu, Department of Earth and Atmospheric Sciences, NIT Rourkela, Rourkela-769008, India, ([rilbhaskar@gmail.com](mailto:rilbhaskar@gmail.com))

This supporting file contains

Figure S1-S5

**Supporting figures:**

**
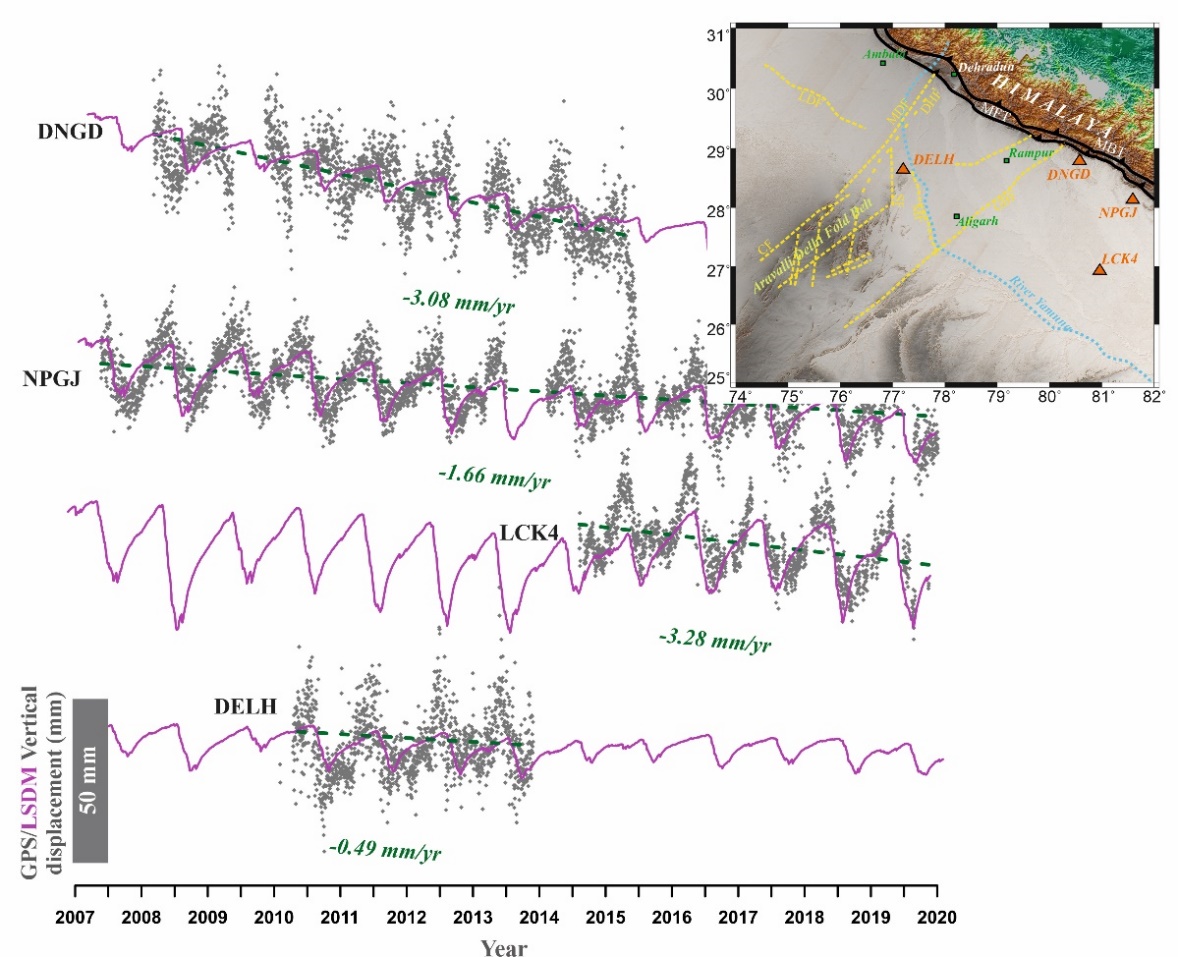
**

**Figure. S1: GPS and LSDM vertical displacement time series from Delhi and surrounding regions.** Representative GPS and LSDM vertical time series from Delhi and surrounding regions (marked in the inset map). The vertical displacements show surface subsidence (indicated by negative trends) due to groundwater extraction. This figure was generated using Grapher graphical application (version 16.6.478 URL: <https://www.goldensoftware.com/products/grapher>) and Generic Mapping Tools (version 6.3.0; URL: <https://www.generic-mapping-tools.org/download/>).


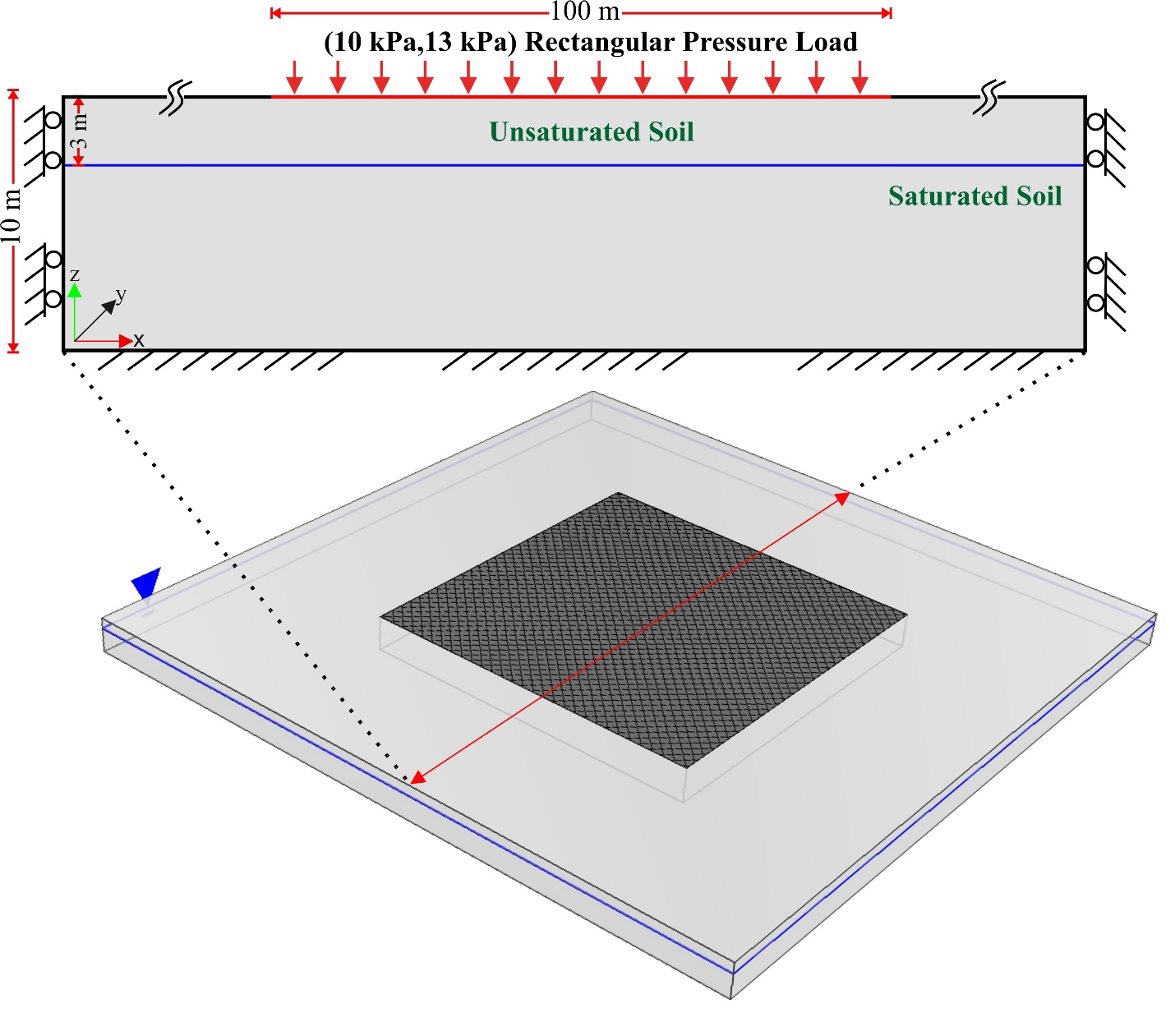


**Figure. S2: Settle3D Model boundary.** The cross-sectional view (top panel) represents the model boundary condition on the 3D model (bottom panel) used for settlement analysis. The bottom boundary is fixed, while all four side boundaries, located at a large distance from the model center, have the rolling boundary condition. A Rectangular (100 m^2^) Area Pressure Load (10 kPa and 13 kPa) is applied over the soil surface. The blue line in the top panel represents the zone of transition between saturated and unsaturated soil intervals. This figure was generated using Settle 3D (version 2.0 URL: <https://www.rocscience.com/software/settle3>) and Corel Draw graphical application (version 22.2.0.532 URL: <https://www.coreldraw.com/en>).

**
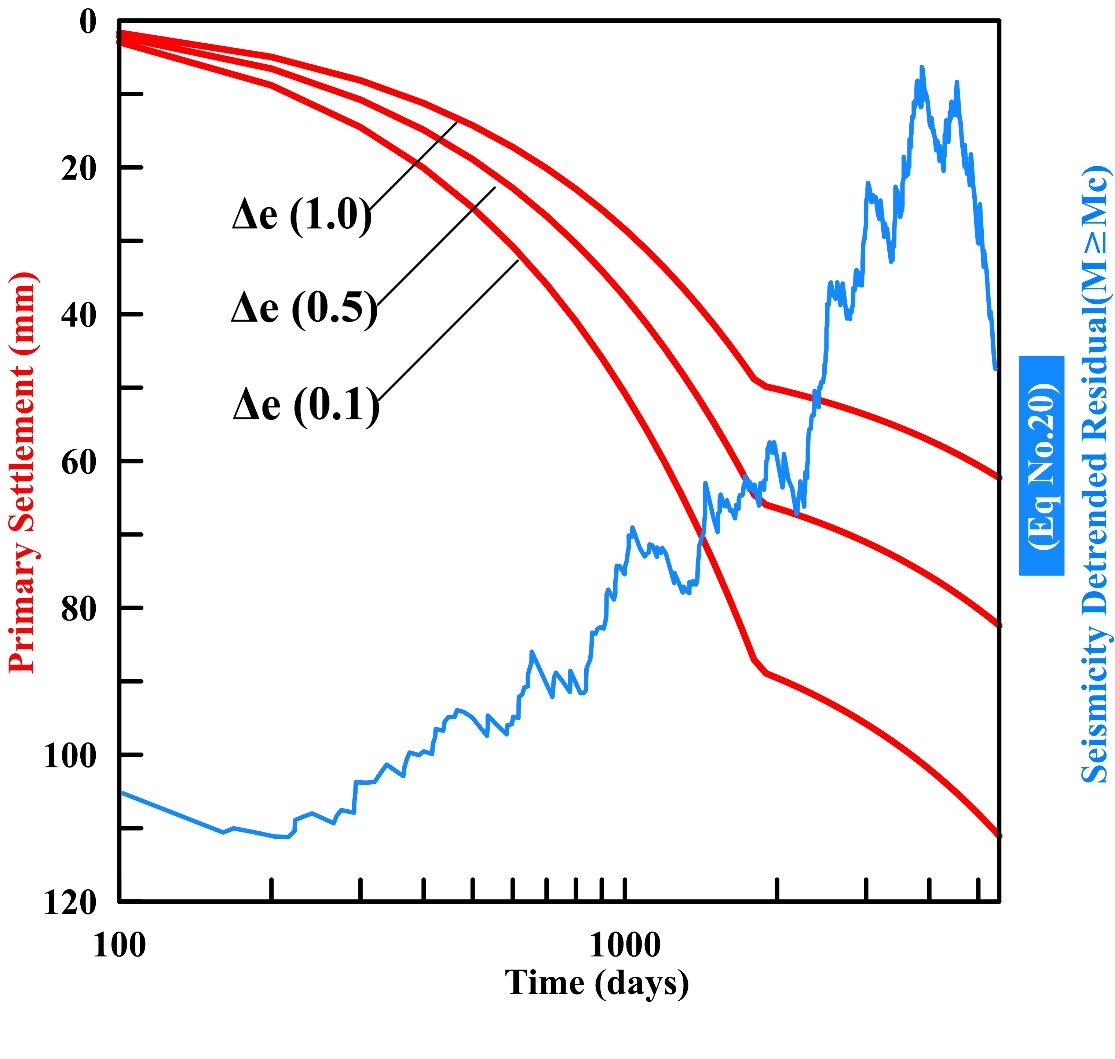
**

**Figure. S3: Correlation analysis between settlement and detrended residual seismicity.** Seismicity detrended residual (M≥Mc) of the Aravali Delhi fold belt region and the primary settlement of the soil layer with void ratio values (1.0, 0.5, 0.1) for the 2000-2015 period, where day 1 is considered as 1^st^ of January 2000. This figure was generated using Grapher graphical application(version 16.6.478 URL: <https://www.goldensoftware.com/products/grapher>).

**
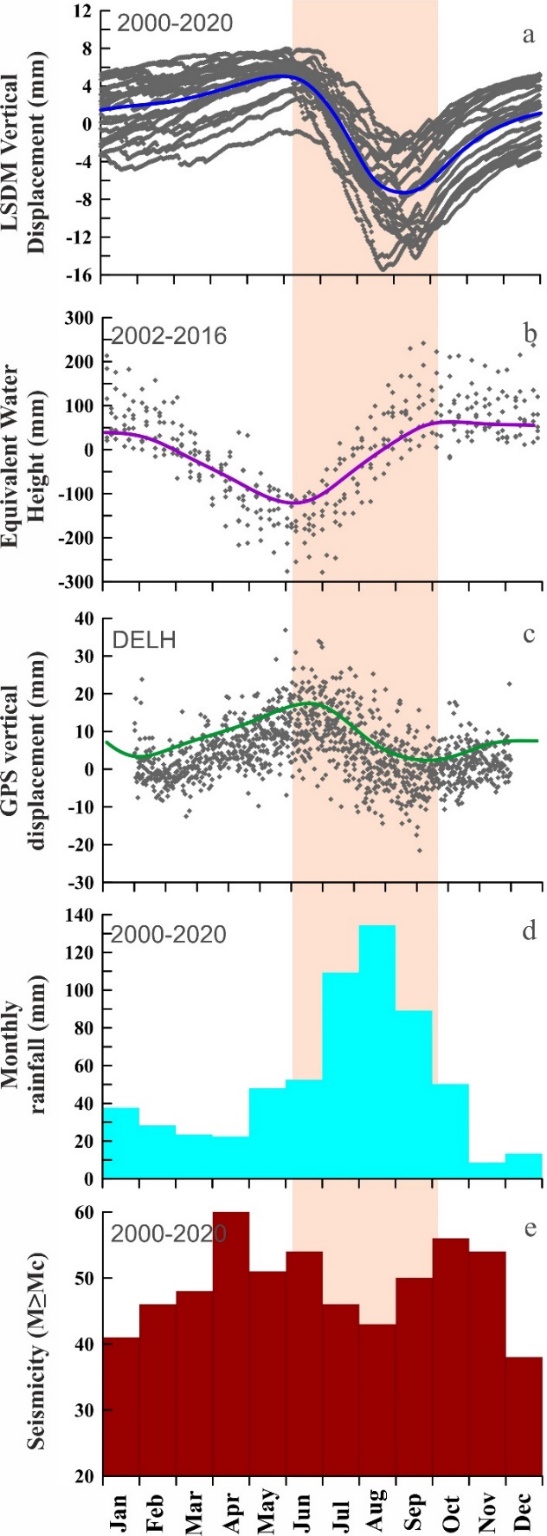
**

**Figure. S4:** **Monthly Stack Time Series analysis of various geophysical parameters.** Correlation between LSDM-derived vertical displacement, Equivalent Water Height, GPS-derived vertical displacement, rainfall, and seismicity frequency from the Aravalli Delhi fold belt. Figure (a-e) represents monthly stacked time series of LSDM vertical displacement, equivalent water height, GPS vertical displacement (station DELH), rainfall derived from TRMM, and monthly seismicity (M$\geq Mc$). The orange-colored vertical strip drawn across the panels represents the hydrological loading period. This figure was generated using Grapher graphical application (version 16.6.478 URL: <https://www.goldensoftware.com/products/grapher>).


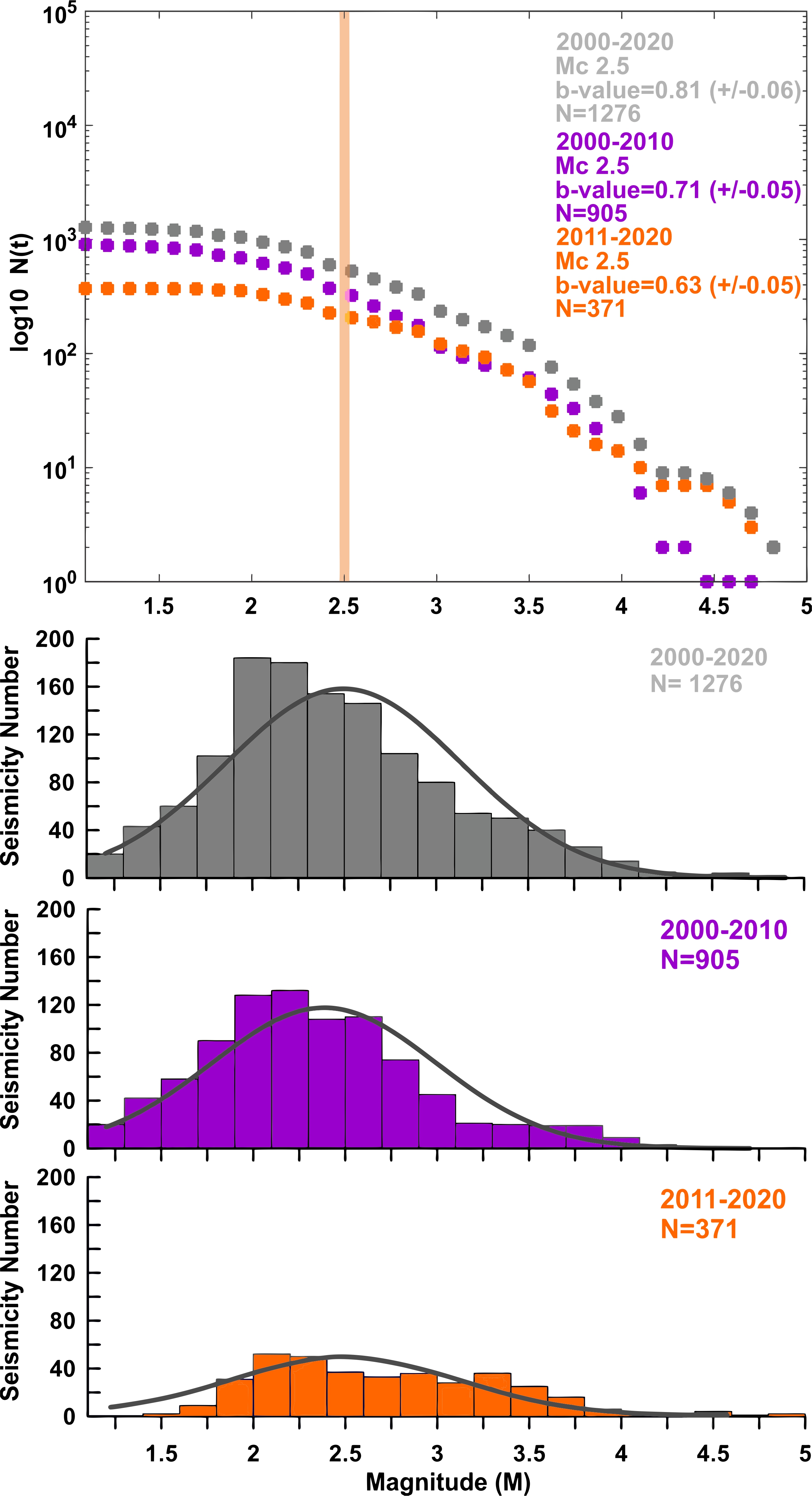


**Figure. S5: Magnitude completeness (Mc) of seismicity over the Delhi region with different time periods.** Note that the overall Mc is constant i.e., 2.5 over different time periods. This figure was generated using MATLAB-Mathworks (version R2018a 9.5.0.9444 URL: <https://in.mathworks.com/products/matlab.html>) and Grapher graphical application(version 16.6.478 URL: <https://www.goldensoftware.com/products/grapher>).
